# Using excess deaths and testing statistics to determine COVID-19 mortalities

**DOI:** 10.1007/s10654-021-00748-2

**Published:** 2021-05-17

**Authors:** Lucas Böttcher, Maria R. D’Orsogna, Tom Chou

**Affiliations:** 1 Dept. of Computational Medicine, UCLA, Los Angeles, CA 90095-1766 USA; 2 Computational Social Science, Frankfurt School of Finance and Management, Frankfurt am Main, 60322 Germany; 3 Dept. of Mathematics, California State University at Northridge, Los Angeles, CA 91330-8313 USA; 4 Dept. of Mathematics, UCLA, Los Angeles, CA 90095-1555 USA

**Keywords:** COVID-19, Excess deaths, Mortality, Test statistics

## Abstract

Factors such as varied definitions of mortality, uncertainty in disease prevalence, and biased sampling complicate the quantification of fatality during an epidemic. Regardless of the employed fatality measure, the infected population and the number of infection-caused deaths need to be consistently estimated for comparing mortality across regions. We combine historical and current mortality data, a statistical testing model, and an SIR epidemic model, to improve estimation of mortality. We find that the average excess death across the entire US from January 2020 until February 2021 is 9$\%$ higher than the number of reported COVID-19 deaths. In some areas, such as New York City, the number of weekly deaths is about eight times higher than in previous years. Other countries such as Peru, Ecuador, Mexico, and Spain exhibit excess deaths significantly higher than their reported COVID-19 deaths. Conversely, we find statistically insignificant or even negative excess deaths for at least most of 2020 in places such as Germany, Denmark, and Norway.

**Supplementary Information:**

The online version contains supplementary material available at 10.1007/s10654-021-00748-2.

## Introduction

The novel severe acute respiratory syndrome coronavirus 2 (SARS-CoV-2) first identified in Wuhan, China in December 2019 quickly spread across the globe, leading to the declaration of a pandemic on March 11, 2020 [1]. As of April 2021, an estimated 130 million people have been infected with COVID-19 disease, with more than 2.8 million deaths across 219 countries [2]. About 104 million people have recovered globally. Properly ascertaining the severity of any infectious disease is crucial for identifying near-future scenarios, and designing intervention strategies. This is especially true for SARS-CoV-2 given the relative ease with which it spreads, due to long incubation periods, asymptomatic carriers, and stealth transmissions [3]. Most measures of severity are derived from the number of deaths, the number of confirmed and unconfirmed infections, and the number of secondary cases generated by a single primary infection, to name a few. Estimating these quantities, determining how they evolve in a population, and how they are to be compared across groups, and over time, is challenging due to many confounding variables and uncertainties [4].

For example, quantifying COVID-19 deaths across jurisdictions must take into account the existence of different protocols in assigning cause of death, cataloging co-morbidities [5], and lag-time reporting. Inconsistencies also arise in the way deaths are recorded, especially when COVID-19 is not the direct cause of death, rather a co-factor leading to complications such as pneumonia and other respiratory ailments [6]. In Italy, the clinician’s best judgment is called upon to classify the cause of death of an untested person who manifests COVID-19 symptoms. In some cases, such persons are given postmortem tests, and if results are positive, added to the statistics. Criteria vary from region to region [7]. In Germany, postmortem testing is not routinely employed, possibly explaining the large difference in mortality between the two countries at the onset of the pandemic. In the US, if typical symptoms are observed, the patient’s death can be registered as due to COVID-19 even without a positive test [8]. Certain jurisdictions will list dates on which deaths actually occurred, others list dates on which they were reported, leading to potential lag-times. Other countries tally COVID-19 related deaths only if they occur in hospital settings, while others also include those that occur in private and/or nursing homes.

In addition to the difficulty in obtaining accurate and uniform fatality counts, estimating the prevalence of the disease is also a challenging task. Large-scale testing of a population where a fraction of individuals is infected, relies on unbiased sampling, reliable tests, and accurate recording of results. One of the main sources of systematic bias arises from the tested subpopulation: due to shortages in testing resources, or in response to public health guidelines, COVID-19 tests have more often been conducted on symptomatic persons, and the elderly. Such non-random testing may lead to overestimation the infected fraction of the population.

Different types of tests also probe different infected subpopulations. Tests based on reverse-transcription polymerase chain reaction (RT–PCR), whereby viral genetic material is detected primarily in the upper respiratory tract and amplified, probe individuals who are actively infected. Serological tests (such as enzyme-linked immunosorbent assay, ELISA) detect antiviral antibodies and thus measure individuals who have been infected, including those who have recovered.

Finally, different types of tests exhibit significantly different “Type I” (false positive) and “Type II” (false negative) error rates, $\mathrm{FPR}$ and $\mathrm{FNR}$, respectively. The accuracy of RT–PCR tests depends on viral load which may be too low to be detected in individuals at the early stages of the infection, and may also depend on which sampling site in the body is chosen. Within serological testing, the kinetics of antibody response and antibody waning are still largely unknown [9, 10]. Instrumentation errors and sample contamination may also result in a considerable number of false positives and/or false negatives. Specifically, at low prevalence, Type I false positive errors can significantly bias the estimation of fatality measures.

Other quantities that are useful in tracking the dynamics of a pandemic include the number of recovered individuals, tested, or untested. These quantities may not be easily inferred from data and need to be estimated from fitting mathematical models such as SIR-type ODEs [11], age-structured PDEs [12], or network/contact models [13–15].

Administration of tests and estimation of all quantities above can vary widely across jurisdictions, making it difficult to properly compare numbers across them. In this paper, we incorporate excess death data, testing statistics, and mathematical modeling to self-consistently compute and compare mortality across different jurisdictions. In particular, we will use excess mortality statistics [16–18] to infer the number of COVID-19-induced deaths in different regions. We then present a statistical testing model to estimate jurisdiction-specific infected fractions and mortalities, their uncertainty, and their dependence on testing bias and errors. Our statistical analyses and source codes are available at [19].

## Methods

### Mortality measures

Different fatality rate measures have been used to quantify epidemic outbreaks [20]. One of the most common is the case fatality ratio ($\mathrm{CFR}$) defined as the ratio between the number of confirmed “infection-caused” deaths $D_{\mathrm{c}}$ in a specified time window and the number of infections $N_{\mathrm{c}}$ confirmed within the same time window, $\mathrm{CFR} = D_{\mathrm{c}}/N_\mathrm{c}$ [21]. Depending on how deaths $D_{\mathrm{c}}$ are counted and how infected individuals $N_{\mathrm{c}}$ are defined, the operational $\mathrm{CFR}$ may vary and may exceed one if some deaths are not included in $N_{\mathrm{c}}$.

Another frequently used measure is the infection fatality ratio ($\mathrm{IFR}$) defined as the true number of “infection-caused” deaths $D = D_\mathrm{c} + D_{\mathrm{u}}$ divided by the actual number of cumulative infections to date, $N_{\mathrm{c}} + N_{\mathrm{u}}$. Here, $D_{\mathrm{u}}$ is the number of unreported infection-caused deaths within a specified period, and $N_{\mathrm{u}}$ denotes the untested or unreported infections during the same period. Thus, $\mathrm{IFR}= D/(N_{\mathrm{c}}+N_{\mathrm{u}})$.

One major limitation of both $\mathrm{CFR}$ and $\mathrm{IFR}$ is that they do not account for the time delay between infection and resolution. Both measures may be quite inaccurate, especially early in an outbreak when the number of cases grows faster than the number of deaths and recoveries [12]. An alternative measure that avoids case-resolution delays is the confirmed resolved mortality $M=D_{\mathrm{c}}/(D_{\mathrm{c}}+R_{\mathrm{c}})$ [12], where $R_{\mathrm{c}}$ is the cumulative number of confirmed recovered cases evaluated in the same specified time window over which $D_{\mathrm{c}}$ is counted. One may also define the true resolved mortality via ${\mathcal {M}}= D/(D + R)$, the proportion of the actual number of deaths relative to the total number of deaths and recovered individuals during a specified time period. If we decompose $R = R_{\mathrm{c}} + R_{\mathrm{u}}$, where $R_{\mathrm{c}}$ and $R_{\mathrm{u}}$ are the confirmed and unreported recovered cases, ${\mathcal {M}}= (D_\mathrm{c}+D_{\mathrm{u}})/(D_{\mathrm{c}}+D_{\mathrm{u}} + R_{\mathrm{c}}+R_{\mathrm{u}})$. The confirmed quantities are related through the total confirmed population $N_{\mathrm{c}} = D_{\mathrm{c}} + R_{\mathrm{c}} + I_{\mathrm{c}}$, where $I_{\mathrm{c}}$ the number of living confirmed infecteds. Applying these definitions to any specified time period (typically from the “start” of an epidemic to the date with the most recent case numbers), we observe that $\mathrm {CFR} \le M$ and $\mathrm {IFR} \le {{{\mathcal {M}}}}$. After the epidemic has long past, when the number of currently infected individuals *I* approaches zero, the two fatality ratios and mortality measures converge if the component quantities are defined and measured consistently, $\lim _{t \rightarrow \infty } \mathrm {CFR}(t) = \lim _{t \rightarrow \infty } M(t)$ and $\lim _{t \rightarrow \infty }\mathrm {IFR}(t) = \lim _{t \rightarrow \infty } {\mathcal {M}}(t)$ [12].

**Table 1:** Definitions of mortality measures. Quantities with subscript “c” and “u” denote confirmed (*i.e.*, positively tested) and unconfirmed populations. For instance, $D_{\mathrm{c}}$, $R_{\mathrm{c}}$, and $N_{\mathrm{c}}$ denote the total number of confirmed dead, recovered, and infected individuals, respectively. $d^{(j)}(i)$ is the number of individuals who have died in the $i{\mathrm{th}}$ time window (*e.g.*, day, week) of the $j{\mathrm{th}}$ previous year. The mean number of excess deaths ${\bar{D}}_{\mathrm{e}}$ within all periods $i=1,\ldots , k$ is thus $\sum _{i=1}^{k} \left[ d^{(0)}(i)- {\frac{1}{J}}\sum _{j=1}^{J} d^{(j)}(i)\right]$. The total number of infection-caused deaths $D_{\mathrm{c}}+D_{\mathrm{u}}$ can be estimated using the excess deaths $D_{\mathrm{e}}$ as detailed in the main text. We have also included raw death numbers/100,000 and the mean excess deaths *r* relative to the mean number of deaths over the same period of time from past years [see Eq. (1)]

Subpopulation	Measure *Z*		
	Fatality Ratios	Resolved Mortality	Excess Death Indices
Confirmed	$\displaystyle { \mathrm{CFR}= \frac{D_{\mathrm{c}}}{N_{\mathrm{c}}}}$	$\displaystyle M=\frac{D_{\mathrm{c}}}{D_{\mathrm{c}}+R_{\mathrm{c}}}$	$D_{\mathrm{e}} \text{per 100,000}:\displaystyle { \frac{D_{\mathrm{c}}+D_{\mathrm{u}}}{100,000}}$
Total	$\displaystyle \mathrm{IFR}=\frac{D_{\mathrm{c}} + D_{\mathrm{u}}}{ N_{\mathrm{c}} + N_{\mathrm{u}}}$	$\displaystyle {{{\mathcal {M}}}}=\frac{D_{\mathrm{c}}+D_{\mathrm{u}}}{D_{\mathrm{c}} + D_{\mathrm{u}} + R_{\mathrm{c}} + R_{\mathrm{u}}}$	Relative: $\displaystyle {\bar{r}} = \frac{\sum _{i}\left[ d^{(0)}(i) - {\frac{1}{J}} \sum _{j}^{J} d^{(j)}(i)\right] }{{\frac{1}{J}}\sum _{j}^{J} \sum _{i}d^{(j)}(i)}$

The mathematical definitions of the four basic mortality measures $Z = \mathrm{CFR, IFR}, M, {{{\mathcal {M}}}}$ defined above are given in Table 1 and fall into two categories, confirmed and total. Confirmed measures ($\mathrm{CFR}$ and *M*) rely only on positive test counts, while total measures ($\mathrm{IFR}$ and ${{{\mathcal {M}}}}$) rely on projections to estimate the number of infected persons in the total population *N*. Of the measures listed in Table 1, the fatality ratio $\mathrm{CFR}$ and confirmed resolved mortality *M* do not require estimates of unreported infections, recoveries, and deaths and can be directly derived from the available confirmed counts $D_\mathrm{c}$, $N_{\mathrm{c}}$, and $R_{\mathrm{c}}$ [22]. Estimation of $\mathrm {IFR}$ and the true resolved mortality ${{\mathcal {M}}}$ requires the additional knowledge on the unconfirmed quantities $D_{\mathrm{u}}$, $N_{\mathrm{u}}$, and $R_{\mathrm{u}}$. We now describe the possible ways to estimate these quantities, along with the associated sources of bias and uncertainty.

### Excess death data

An unbiased way to estimate $D = D_{\mathrm{c}} + D_{\mathrm{u}}$, the cumulative number of deaths, is to compare total deaths within a time window in the current year to those in the same time window of previous years, before the pandemic.

**Fig. 1:**
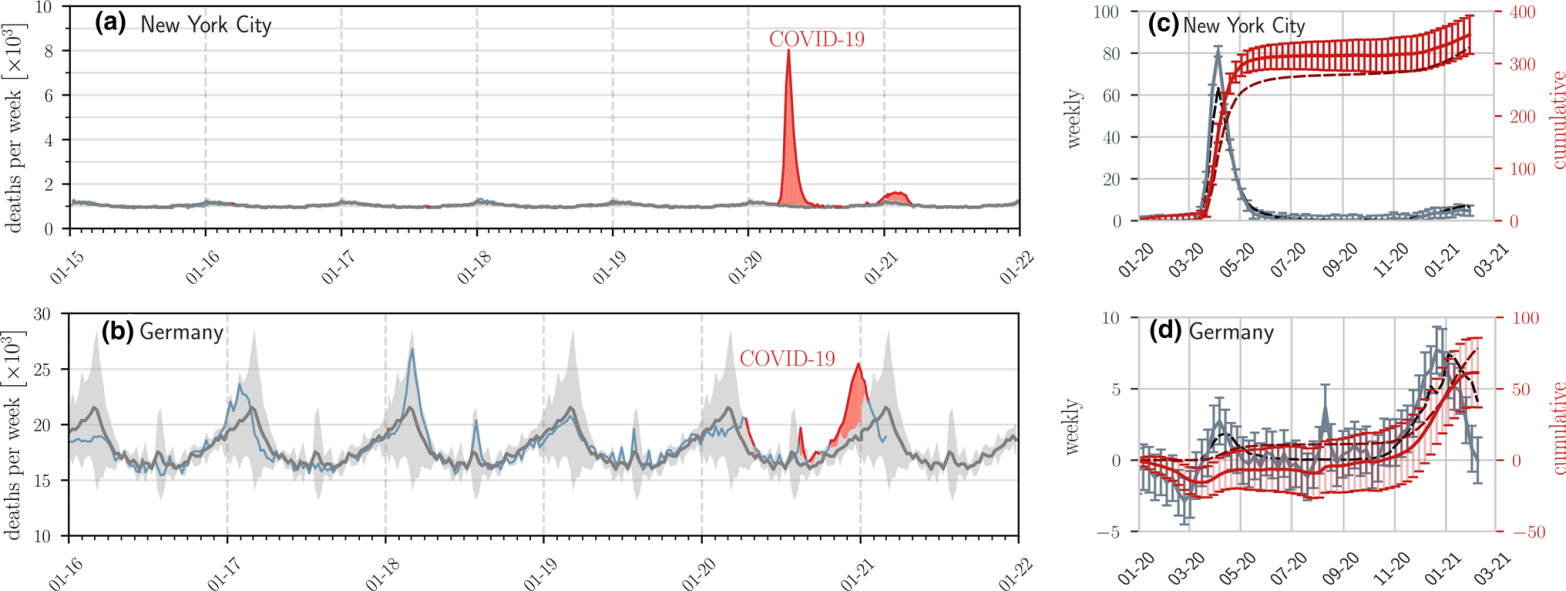
Examples of seasonal mortality and excess deaths. The evolution of weekly deaths in (a) NYC (over seven years) and (b) Germany (over six years) derived from data in Refs. [28, 29]. Grey solid lines and shaded regions represent the historical numbers of deaths and corresponding confidence intervals defined in Eq. (1). Blue solid lines indicate weekly deaths, and weekly deaths that lie outside the confidence intervals are indicated by solid red lines. The red shaded regions represent statistically significant mean cumulative excess deaths ${\bar{D}}_{\mathrm{e}}$. The reported weekly confirmed deaths $d^{(0)}_{\mathrm{c}}(i)$ (dashed black curves), reported cumulative confirmed deaths $D_{\mathrm{c}}(k)$ (dashed dark red curves), weekly excess deaths ${\bar{d}}_{\mathrm{e}}(i)$ (solid grey curves), and cumulative excess deaths ${\bar{D}}_{\mathrm{e}}(k)$ (solid red curves) are plotted in units of per 100,000 in (c) and (d) for NYC and Germany, respectively. The excess deaths and the associated 95% confidence intervals given by the error bars are constructed from historical death data in (a-b) and defined in Eqs. (1) and (2). In NYC there is clearly a significant number of excess deaths that can be safely attributed to COVID-19, while in the first half of 2020 there had been no significant excess deaths in Germany. Excess death data from other jurisdictions are shown in the SI and typically show excess deaths greater than reported confirmed deaths [with Germany an exception as shown in (d)]

Since COVID-19 exhibits appreciable fatality, one may reasonably expect that most “excess” deaths can be attributed to the pandemic [23–27]. Within each affected region, these excess deaths $D_{\mathrm{e}}$ relative to “historical” deaths, are independent of testing limitations and do not suffer from highly variable definitions of COVID-induced death. Thus, $D_{\mathrm{e}}$ is a more inclusive measure of virus-induced deaths than $D_{\mathrm{c}}$ and can be used to estimate the total number of deaths, $D_{\mathrm{e}} \approx D_{\mathrm{c}} + D_{\mathrm{u}}$. Moreover, using data from multiple past years, one can also estimate the uncertainty in $D_{\mathrm{e}}$. In practice, deaths are tallied daily, weekly [23, 30], or in monthly aggregates [29, 31] with historical records dating back *J* years so that for every period *i* there are a total of $J+1$ values. We denote by $d^{(j)}(i)$ the total number of deaths recorded in period *i* from the $j^{\mathrm{th}}$ previous year where $0 \le j \le J$ and where $j=0$ indicates the current year. To quantify the total cumulative excess deaths we define $d_\mathrm{e}^{(j)}(i)=d^{(0)}(i)-d^{(j)}(i)$ as the excess deaths in period *i* relative to that of the $j^{\mathrm{th}}$ previous year. Since $d^{(0)}(i)$ is the total number of deaths in week *i* of the current year, by definition $d_{\mathrm{e}}^{(0)}(i) \equiv 0$. The excess deaths during week *i* averaged over *J* past years, ${\bar{d}}_{\mathrm{e}}(i)$, and the associated, unbiased variance $\sigma _\mathrm{e}(i)$ [32] are given by

1 $$\begin{aligned} \begin{aligned} {\bar{d}}_{\mathrm{e}}(i)&= {1\over J}\sum _{j=1}^{J} d_{\mathrm{e}}^{(j)}(i), \\ \sigma _{\mathrm{e}}^{2}(i)&= {1\over J-1}\sum _{j=1}^{J} \left[ d_{\mathrm{e}}^{(j)}(i)-{\bar{d}}_{\mathrm{e}}(i)\right] ^{2}. \end{aligned} \end{aligned}$$

The corresponding quantities accumulated over *k* weeks define the mean and variance of the cumulative excess deaths ${\bar{D}}_{\mathrm{e}}(k)$ and $\Sigma _{\mathrm{e}}(k)$:

2 $$\begin{aligned} \begin{aligned} {\bar{D}}_{\mathrm{e}}(k)&= {1\over J}\sum _{j=1}^{J} \sum _{i=1}^k d_{\mathrm{e}}^{(j)}(i)=\sum _{i=1}^k {\bar{d}}_{\mathrm{e}}(i), \\ \Sigma _{\mathrm{e}}^{2}(k)&= \frac{1}{J-1}\sum _{j=1}^J\sum _{i=1}^k \left[ d_{\mathrm{e}}^{(j)}(i)-{\bar{d}}_{\mathrm{e}}(i)\right] ^{2}=\sum _{i=1}^k \sigma _{\mathrm{e}}^{2}(i), \\ \end{aligned} \end{aligned}$$

where deaths and their variances are accumulated from the first to the $k^{\mathrm{th}}$ week of the pandemic. The variance in Eqs. (1) and (2) arise from the variability in the number of deaths from the same time period across *J* previous years.

We gathered excess death statistics from over 75 countries and all US states. Some of the data derive from open-source online repositories as listed by official statistical bureaus and health ministries [23–27, 31]; other data are elaborated and tabulated in Ref. [29]. In some countries excess death statistics are available only for a limited number of states or jurisdictions (*e.g.*, Turkey and Indonesia). The US death statistics that we use in this study is based on weekly death data between 2015–2019 [31]. For all other countries, the data collection periods are summarized in Ref. [29]. Fig. 1(a-b) shows historical death data for New York City (NYC) and Germany, while Fig. 1(c-d) plots the associated confirmed and excess deaths and their confidence levels computed from Eqs. (1) and (2). We assumed that the cumulative summation is performed from the start of 2020 to the second week of February 2021 (week number $k=7$). Significant numbers of excess deaths are clearly evident for NYC, while Germany had not experienced significant excess COVID-19 deaths in the first half of 2020.

To evaluate $\mathrm{CFR}$ and *M*, only data on $D_{\mathrm{c}}$, $N_\mathrm{c}$, and $R_{\mathrm{c}}$ are required, which are are tabulated by many jurisdictions. We can approximate the numerators of the $\mathrm{IFR}$ and ${{{\mathcal {M}}}}$ by using, for example, the mean excess deaths ${\bar{D}}_{\mathrm{e}}\approx D_{\mathrm{c}}+D_{\mathrm{u}}$ defined in Eq. (2). For the denominators, estimates of the unconfirmed infected $N_{\mathrm{u}}$ and unconfirmed recovered populations $R_{\mathrm{u}}$ are required. In the next two sections we propose methods to estimate $N_{\mathrm{u}}$ using a statistical testing model and $R_\mathrm{u}$ using a compartmental population model.

### Statistical testing model with bias and testing errors

Testing bias and different sources of uncertainty in disease testing confound the estimation of the true prevalence of a disease. If not corrected, a sampling bias due to preferential testing of symptomatic individuals, healthcare workers, and certain high-risk groups [33] may lead to an overestimation of the fraction or total number of infected individuals. Since the total number of confirmed and unconfirmed cases, $N_{\mathrm{c}} + N_{\mathrm{u}}$, appears in the denominator of the $\mathrm{IFR}$, we develop a statistical model for its estimation in the presence of testing errors and bias in administration of tests.

Although $N_{\mathrm{c}} + N_{\mathrm{u}}$ used to estimate the $\mathrm{IFR}$ includes those who have died, it may or may not include those who have recovered. If *S*, *I*, *R*, *D* are the numbers of susceptible, currently infected, recovered, and deceased individuals, the total population is $N =S + I + R + D$ and the infected fraction can be defined as $f = (N_{\mathrm{c}} + N_{\mathrm{u}})/N=(I + R + D)/N$ for tests that include recovered and deceased individuals (*e.g.*, antibody tests), or $f = (N_{\mathrm{c}} + N_{\mathrm{u}})/N=(I+D)/N$ for tests that only count currently infected individuals (*e.g.*, RT–PCR tests). If we assume that the total population *N* can be inferred from census estimates, the problem of identifying the number of unconfirmed infected persons $N_{\mathrm{u}}$ is mapped onto the problem of identifying the true fraction *f* of the population that has been infected. Thus, we need to estimate *f* from testing results.

**Fig. 2:**
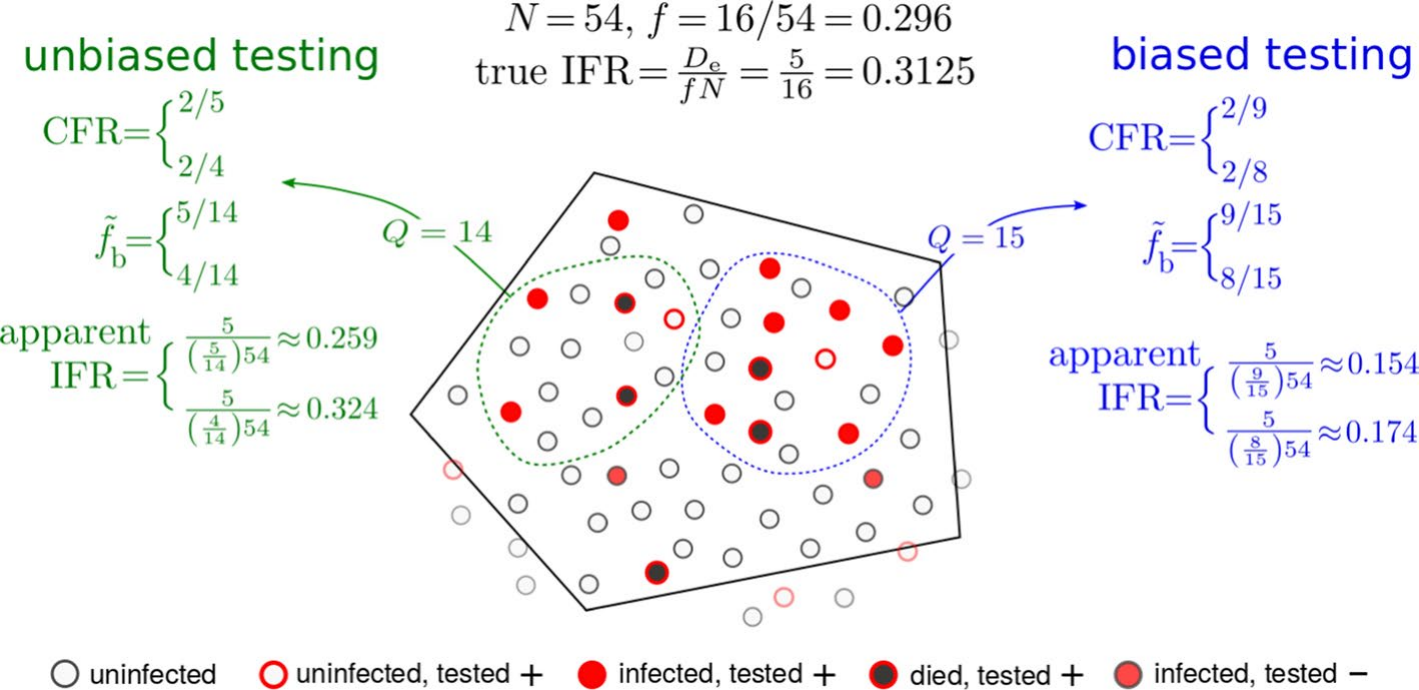
Biased and unbiased testing of a population. A hypothetical scenario of testing a total population of $N=54$ individuals within a jurisdiction (solid black boundary). Filled red circles represent the true number of infected individuals who tested positive and the black-filled red circles indicate individuals who have died from the infection. Open red circles denote uninfected individuals who were tested positive (false positives) while filled red circles with dark gray borders are infected individuals who were tested negative (false negatives). In the jurisdiction of interest, five have died of the infection while 16 are truly infected. The true fraction *f* of infected in the entire population is thus $f=16/54$ and the true $\mathrm{IFR}=5/16$. However, under testing (green and blue) samples, a false positive is shown to arise. If the measured positive fraction ${\tilde{f}}_{\mathrm{b}}$ is derived from a biased sample (blue), the estimated *apparent* $\mathrm {IFR}$ can be quite different from the true one. For a less biased (more random) testing sample (green), a more accurate estimate of the total number of infected individuals is $N_{\mathrm{c}}+N_{\mathrm{u}} = {\tilde{f}}_{\mathrm{b}} N = (5/14)\times 54 \approx 19$ when the single false positive in this sample is included, and ${\tilde{f}}_{\mathrm{b}} N = (4/14)\times 54 \approx 15$ when the single false positive case is excluded, and allows us to more accurately infer the $\mathrm{IFR}$. Note that $\mathrm{CFR}$ is defined according to the tested quantities $D_{\mathrm{c}}/N_{\mathrm{c}}$ which are precisely 2/9 and 2/5 for the blue and green sample, respectively, if false positives are considered. When false negatives are known and factored out $\mathrm {CFR}=2/8$ and 2/4, for the blue and green samples, respectively

Figure 2 shows a schematic of a hypothetical initial total population of $N=54$ individuals in a specified jurisdiction. Without loss of generality we assume there are no unconfirmed deaths, $D_{\mathrm{u}} = 0$, and that all confirmed deaths are equivalent to excess deaths, so that ${\bar{D}}_{\mathrm{e}} = D_{\mathrm{c}} = 5$. Apart from the number of deceased, we also show the number of true infected and uninfected subpopulations and label them as true positives, false positives, and false negatives. The true number of infected individuals is $N_{\mathrm{c}} + N_{\mathrm{u}} = 16$ which yields the true fraction $f = 16/54 = 0.296$ and an $\mathrm{IFR} = 5/16 = 0.312$.

Also shown in Fig. 2 are two examples of sampling. Biased sampling and testing are depicted by the blue contour in which 6 of the 15 individuals are alive and infected, 2 are deceased, and the remaining 7 are healthy. For simplicity, we start by assuming no testing errors. The measured infected fraction of this sample, ${\tilde{f}}_{\mathrm{b}}= 8/15 = 0.533 > f = 0.296$, is biased since it includes a higher proportion of infected persons than that of the entire jurisdiction. Using this biased measured fraction yields the apparent $\mathrm{IFR} = 5/(0.533\times 54) \approx 0.174$, which significantly underestimates the true $\mathrm{IFR} \approx 0.312$. A less-biased sample, shown by the green contour, yields an infected fraction of $4/14 \approx 0.286$ and an apparent $\mathrm{IFR}=5/(0.286\times 54)\approx 0.324$ which are much closer to the true fraction $f=0.296$ and the true, “correct” $\mathrm{IFR}\approx 0.312$.

In both samples discussed above we neglected testing errors such as false positives, as indicated in Fig. 2. Tests that are unable to distinguish false positives from true negatives would yield a larger $N_{\mathrm{c}}$, resulting in an apparent infected fraction ${\tilde{f}}_{\mathrm{b}}=9/15$ and an even smaller apparent $\mathrm{IFR}\approx 0.154$, as in the blue sample. By contrast, the false positive testing errors in the green sample would yield an apparent infected fraction ${\tilde{f}}_{\mathrm{b}}=5/15 \approx 0.333$ and $\mathrm{IFR} \approx 0.259$. Given a true infected fraction *f*, we now derive the probability of measuring the value ${\tilde{f}}_{\mathrm{b}}$ under biased testing and testing errors rates $\mathrm{FPR}$ and $\mathrm{FNR}$.

We begin by proposing a parametric form for the apparent or measured (under biased testing) infected fraction

3 $$\begin{aligned} f_{\mathrm{b}}\equiv f_{\mathrm{b}}(f, b)\equiv {fe^{b} \over f(e^{b}-1)+1}. \end{aligned}$$

Equation (3) allows us to connect $f_{\mathrm{b}}$ to the true infection fraction *f*. The bias parameter $-\infty< b < \infty$ in Eq. (3) describes how an infected or uninfected individual might be preferentially selected for testing, with $b <0$ (and $f_\mathrm{b}< f$) indicating under-testing of infected individuals, and $b>0$ (and $f_{\mathrm{b}}> f$) representing over-testing of infecteds. A truly random, unbiased sampling arises only when $b=0$ and $f_{\mathrm{b}} = f$. Given *Q* administered tests to date, the number of recorded positive tests ${\tilde{Q}}^{+}$, error rates $\mathrm{FPR}, \mathrm{FNR}$, and true infected fraction *f*, we derive in the Supplementary Information (SI) the likelihood of observing a positive fraction ${\tilde{f}}_{\mathrm{b}}\!:={\tilde{Q}}^{+}/Q$

4 $$\begin{aligned} P({\tilde{f}}_{\mathrm{b}}\vert f, \theta _{\mathrm{T}})\approx {1\over \sqrt{2\pi }\sigma _{\mathrm{T}}} \exp \left[ -{({\tilde{f}}_{\mathrm{b}} -\mu )^{2}\over 2\sigma _{\mathrm{T}}^{2}}\right] , \end{aligned}$$

in which

5 $$\begin{aligned} \begin{aligned} \mu&\equiv f_{\mathrm{b}}(f,b)(1-\mathrm{FNR}) + (1-f_{\mathrm{b}}(f,b))\mathrm{FPR}, \\ \sigma _{\mathrm{T}}^{2}&\equiv \mu (1-\mu )/Q. \end{aligned} \end{aligned}$$

Here, $\mu$ is the expected value of the measured and biased fraction ${\tilde{f}}_{\mathrm{b}}$, and $\sigma _{\mathrm{T}}^{2}$ is its variance. The parameters associated with testing are denoted $\theta _{\mathrm{T}} =\{Q, b, \mathrm{FPR}, \mathrm{FNR}\}$, and may be time-dependent and may change from sample to sample.

Our formulae Eqs. (3), (4), and (5) provide relationships among the variables and testing parameters. In the testing context, *Q*, ${\tilde{Q}}^{+}$, and thus ${\tilde{f}}_\mathrm{b}={\tilde{Q}}^{+}/Q$ will be measured and used to infer *f*, which we have explicitly separated out in Eq. (4) above. The goal is to use ${\tilde{f}}_{\mathrm{b}}$ as an estimate of $f_{\mathrm{b}}$ and ultimately estimate the true infected fraction *f* through Eq. (3). This requires *a-priori* knowledge of the parameters $\theta _{\mathrm{T}}$. For example, $\mathrm{FPR}$ and $\mathrm{FNR}$ are typically given by test specifications. To estimate *b* instead, one must conduct tests on a small truly random sample and compare results to those from a more widely used biased one. A summary of the main variables that we use in our statistical testing model is given in Table 2.

For a given measured value ${\tilde{f}}_{\mathrm{b}}$, s simple maximum likelihood estimate of *f* can be found by maximizing Eq. (4) with respect to *f*, with all other parameters in $\theta _{\mathrm{T}}$ specified,

6 $$\begin{aligned} {\hat{f}} \approx {{\tilde{f}}_{\mathrm{b}} -\mathrm{FPR} \over e^{b}(1-\mathrm{FNR}-{\tilde{f}}_{\mathrm{b}})+ {\tilde{f}}_{\mathrm{b}}-\mathrm{FPR}}. \end{aligned}$$

Additional correction terms of ${{{\mathcal {O}}}}(1/\sqrt{Q})$ are neglected. Note that although $\mathrm{FNR}$s are typically larger than $\mathrm {FPR}$s, small values of *f* and ${\tilde{f}}_{\mathrm{b}}$ imply that ${\hat{f}}$ and $\mu$ are more sensitive to the $\mathrm{FPR}$, as indicated by Eqs. (5) and (6).

If time series data for ${\tilde{f}}_{\mathrm{b}}$ are available, one can evaluate the estimated testing fractions in Eq. (6) for each time interval. Assuming that serological tests can identify infected individuals long after symptom onset, the latest value of ${\hat{f}}$ would suffice to estimate corresponding mortality metrics such as the $\mathrm {IFR}$. For RT–PCR testing, one generally needs to track how ${\tilde{f}}_{\mathrm{b}}$ evolves in time. A rough estimate would be to use the mean of ${\tilde{f}}_{\mathrm{b}}$ over the whole pandemic period to provide a lower bound of the estimated prevalence ${\hat{f}}$.

**Table 2:** Variables used in statistical testing model. A summary of the main variables that we use in the statistical testing model defined in Eqs. (3)–(6). Above, [0, *N*] and [0, *Q*] denotes a set of integer values, while ${\tilde{f}}_\mathrm{b}:[0,1]$ denotes all rational numbers between 0 and 1. For $f_{\mathrm{b}}, \mathrm{FNR}, \mathrm{FPR}$, [0, 1] represents all real numbers between 0 and 1. It is assumed that $Q, {\tilde{Q}}^{+}$ and ${\tilde{f}}_{\mathrm{b}}\!:={\tilde{Q}}^{+}/Q$ are determined by testing a population of known size *N*

Symbol	Definition
$N\in \mathbb {Z}^{+}$	Population in jurisdiction
*Q* : [0, *N*]	Tests administered
${\tilde{Q}}^+: [0,Q]$	Recorded positive tests
$\displaystyle {f={N_{\mathrm{c}}+N_{\mathrm{u}}\over N}: [0,1]}$	True fraction of infected individuals
$f_{\mathrm{b}}\equiv f_{\mathrm{b}}(f,b): [0,1]$	Infected fraction under biased testing
$\displaystyle {\tilde{f}}_{\mathrm{b}}\!:={{\tilde{Q}}^{+}\over Q}: [0,1]$	Measured infected fraction under biased testing
$b\in \mathbb {R}$	Testing bias
$\mathrm{FPR}: [0,1]$	False positive rate
$\mathrm{FNR}: [0,1]$	False negative rate

The fraction ${\tilde{f}}_{\mathrm{b}}$ measured under biased testing yields only the apparent $\mathrm{IFR} = D_{\mathrm{e}}/({\tilde{f}}_{\mathrm{b}} N)$ or expected apparent $\overline{\mathrm{IFR}} = {\bar{D}}_\mathrm{e}/({\tilde{f}}_{\mathrm{b}} N)$, but Eq. (6) can then be used to estimate the true, corrected $\overline{\mathrm{IFR}} \approx {\bar{D}}_{\mathrm{e}}/({\hat{f}} N)$. For example, under moderate bias $\vert b\vert \lesssim 1$ and assuming $\mathrm{FNR}$, $\mathrm{FPR}$, $\tilde{f}_{\mathrm{{b}}} \lesssim 1$, Eq. (6) can be used to relate the expected true $\overline{\mathrm{IFR}}$ to the expected apparent $\overline{\mathrm{IFR}}$ through ${\bar{D}}_{\mathrm{e}}/({\hat{f}}N) \approx (e^{b}+\mathrm{FPR}/{\hat{f}}){\bar{D}}_{\mathrm{e}}/({\tilde{f}}_{\mathrm{b}}N)$.

Another commonly used representation of the $\mathrm{IFR}$ is $\mathrm {IFR} = p (D_{\mathrm{c}}+D_{\mathrm{u}})/N_{\mathrm{c}} = p D_{\mathrm{e}}/N_\mathrm{c}$. This expression is equivalent to $\mathrm {IFR} = D_{\mathrm{e}}/(f\;N)$ if $p = N_{\mathrm{c}}/(N_{\mathrm{c}}+ N_{\mathrm{u}})\approx {\tilde{Q}}^{+}/(f\;N)$ is defined as the fraction of infected individuals that are confirmed [34, 35]. In this alternative representation, the *p* factor implicitly contains the effects of biased testing. Our approach allows the true infected fraction *f* to be directly estimated from ${\tilde{Q}}^{+}$ and *N*.

While the estimate ${\hat{f}}$ needed to evaluate $\mathrm{IFR}$ depends strongly on *b* and $\mathrm{FPR}$, and weakly on $\mathrm{FNR}$, the *uncertainty* in ${\hat{f}}$ will depend on the uncertainty in the values of *b*, $\mathrm{FPR}$, and $\mathrm{FNR}$. Although statistical models for inferring $\theta _{\mathrm{T}}$ from data can be similarly constructed, here, we use a simple linear approximation to propagate uncertainty in the testing parameters to the squared coefficient of variation $\sigma _{f}^{2}/{\hat{f}}^{2}$ of the estimated infected fraction ${\hat{f}}$. The uncertainties in the mortality indices *Z* decomposed into the uncertainties in their respective individual components are listed in Table 3.

### Using compartmental models to estimate resolved mortalities

Since the number of unreported or unconfirmed recovered individuals $R_{\mathrm{u}}$ required to calculate the total resolved mortality $\mathcal{M}$ is not directly related to excess deaths nor to positive-tested populations, we use an SIR-type compartmental model to relate $R_\mathrm{u}$ to other inferable quantities [11]. Both unconfirmed recovered individuals and unconfirmed deaths $D_{\mathrm{u}}$ are related to unconfirmed infected individuals $I_{\mathrm{u}}$ who recover at rate $\gamma _{\mathrm{u}}$ and die at rate $\mu _{\mathrm{u}}$. The equations for the cumulative numbers of unconfirmed recovered individuals and unconfirmed deaths,

7 $$\begin{aligned} \frac{\text{d}R_{\mathrm{u}}(t)}{\text{d}t} = \gamma _{\mathrm{u}}(t) I_{\mathrm{u}}(t),\qquad \frac{\text{d}D_{\mathrm{u}}(t)}{\text{d}t} = \mu _{\mathrm{u}}(t) I_{\mathrm{u}}(t), \end{aligned}$$

can be directly integrated to find $R_{\mathrm{u}}(t) = \int _{0}^{t} \gamma _{\mathrm{u}}(t')I_{\mathrm{u}}(t')\text{d}t'$ and $D_{\mathrm{u}}(t) = \int _{0}^{t} \mu _{\mathrm{u}}(t')I_{\mathrm{u}}(t')\text{d}t'$. The rates $\gamma _{\mathrm{u}}$ and $\mu _{\mathrm{u}}$ may differ from those averaged over the entire population since testing may be biased towards subpopulations with different values of $\gamma _{\mathrm{u}}$ and $\mu _\mathrm{u}$. If one assumes $\gamma _{\mathrm{u}}$ and $\mu _{\mathrm{u}}$ are approximately constant over the period of interest, we find $R_\mathrm{u}/D_{\mathrm{u}} \approx \gamma _{\mathrm{u}}/\mu _{\mathrm{u}}\equiv \gamma$. We now use $D_{\mathrm{u}} \approx D_{\mathrm{e}} - D_{\mathrm{c}}$ to estimate $R_\mathrm{u} \approx \gamma (D_{\mathrm{e}} - D_{\mathrm{c}})$ and write ${\mathcal {M}}$ as

8 $$\begin{aligned} {\mathcal {M}} =\frac{\displaystyle {D_{\mathrm{e}}}}{D_{\mathrm{e}} + R_{\mathrm{c}} + \gamma (D_{\mathrm{e}}-D_{\mathrm{c}})}. \end{aligned}$$

An estimate for the expected value $\overline{{\mathcal {M}}}$ can be obtained by substituting the excess deaths $D_{\mathrm{e}}$ in Eq. (8) with ${\bar{D}}_{\mathrm{e}}$ from historical data.

Thus, a simple SIR model transforms the problem of determining the number of unreported death and recovered cases in $\overline{{\mathcal {M}}}$ to the problem of identifying the recovery and death rates in the untested population. Alternatively, we can make use of the fact that both the $\overline{\mathrm{IFR}}$ and resolved mortality $\overline{{\mathcal {M}}}$ should have comparable values and match $\overline{{{{\mathcal {M}}}}}$ to $\overline{\mathrm {IFR}}\approx 0.1-1.5$% [35–37] by setting $\gamma \equiv \gamma _{\mathrm{u}}/\mu _{\mathrm{u}}\approx 100-1000$ (see SI for further information). Note that inaccuracies in confirming deaths may give rise to $D_{\mathrm{c}} > {\bar{D}}_{\mathrm{e}}$. Since by definition, infection-caused excess deaths must be greater than the confirmed deaths, we set ${\bar{D}}_{\mathrm{e}}-D_{\mathrm{c}} = 0$ whenever data happens to indicate ${\bar{D}}_{\mathrm{e}}$ to be less than $D_{\mathrm{c}}$.

## Results

Here, we present much of the available worldwide fatality data, construct the excess death statistics, and compute mortalities and compare them across jurisdictions. We show that standard mortality measures significantly underestimate the death toll of COVID-19 for most regions (see Figs. 1, A1, and A2). We also use the data to estimate uncertainties in the mortality measures and relate them to uncertainties of the underlying components and model parameters.

### Excess and confirmed deaths

In NYC, the number of confirmed COVID-19 deaths between March 10, 2020 and December 10, 2020 is 19,694 [38] and thus significantly lower than the 27,938 (95% confidence interval [CI]: 26,516–29,360) reported excess mortality cases [23]. From March 25, 2020 until December 10, 2020, Spain counts 65,673 (99% CI: 91,816–37,061) excess deaths [24], a number that is substantially larger than the officially reported 47,019 COVID-19 deaths [39]. The large difference between excess deaths and reported COVID-19 deaths in Spain and NYC is also observed in Lombardia, one of the most affected regions in Italy. From February 23, 2020 until April 4, 2020, Lombardia reported 8,656 reported COVID-19 deaths [39] but 13,003 (95% CI: 12,335–13,673) excess deaths [27]. Starting April 5 2020, mortality data in Lombardia stopped being reported in a weekly format. In England/Wales, the number of excess deaths from the onset of the COVID-19 outbreak on March 1, 2020 until November 27, 2020 is 70,563 (95% CI: 52,250–88,877) whereas the number of reported COVID-19 deaths in the same time interval is 66,197 [28]. In Switzerland, the number of excess deaths from March 1, 2020 until November 29, 2020 is 5,664 (95% CI: 4,281–7,047) [26], slightly larger than the corresponding 4932 reported COVID-19 deaths [39].

**Fig. 3:**
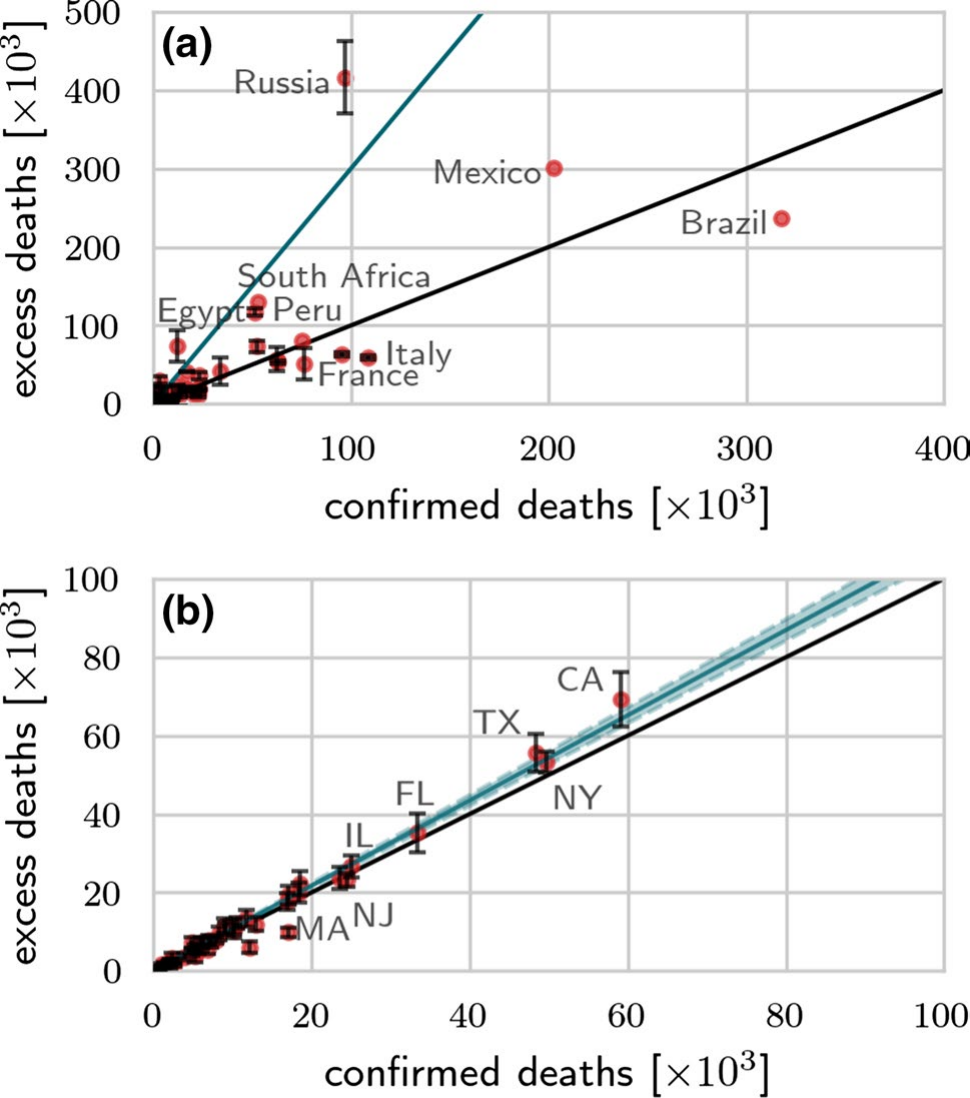
Excess deaths versus confirmed deaths across different countries/states. The number of excess deaths ${\bar{D}}_{\mathrm{e}}$ as of March 30, 2021 [29, 31] (counted from January 2020 onwards) versus confirmed deaths $D_{\mathrm{c}}$ across different countries (a) and US states (b). The black solid lines in both panels have slope 1. In (a) the blue solid line is a guide-line with slope 3; in (b) the blue solid line is a least-squares fit of the data with slope 1.087 (95% CI: 1.052–1.121); blue shaded region). All data were updated on March 30, 2021 [22, 29, 31, 40]

To illustrate the significant differences between excess deaths and reported COVID-19 deaths in various jurisdictions, we plot the excess deaths against confirmed deaths for various countries and US states as of March 30, 2021 in Fig. 3. We observe in Fig. 3a that excess deaths in countries like Egypt, Mexico, Peru, Russia, and South Africa are significantly higher than the corresponding number of confirmed COVID-19 deaths. In particular, they were about three times higher than the number of reported COVID-19 in Egypt, Peru, Russia, and South Africa. For the majority of US states the number of excess deaths is also larger than the number of reported COVID-19 deaths, as shown in Fig. 3b. We performed a least-square fit to calculate the proportionality factor *m* arising in ${\bar{D}}_{\mathrm{e}} = m D_{\mathrm{c}}$ and found $m \approx 1.087$ (95% CI: 1.052–1.121). That is, across all US states, the number of excess deaths is about 9% larger than the number of confirmed COVID-19 deaths in the period from January 2020 until February 2021.

### Estimation of mortality measures and their uncertainties

**Table 3:** Uncertainty propagation for different mortality measures. Table of squared coefficients of variation $\mathrm {CV}^2=\Sigma _{Z}^{2}/Z^{2}$ for the different mortality indices *Z* derived using standard error propagation expansions [41]. Here, we have used the expected values $\overline{\mathrm{IFR}}$ and $\overline{{{\mathcal {M}}}}$ and decomposed the CVs through variances about the mean values of all parameters such as ${\bar{D}}_{\mathrm{e}}$. We use $\Sigma _{N}^{2}, \Sigma _{N_{\mathrm{c}}}^2$, $\Sigma _{R_{\mathrm{c}}}^2$, and $\Sigma _{D_{\mathrm{c}}}^2$ to denote the uncertainties in the total population, confirmed cases, recoveries, and deaths, respectively. The variance of the number of excess deaths is $\Sigma _{\mathrm{e}}^2$, which feature in the $\mathrm{IFR}$ and ${{{\mathcal {M}}}}$. The uncertainty in the infected fraction $\sigma _{f}^{2}$ that contributes to the uncertainty in $\mathrm{IFR}$ depends on uncertainties in testing bias and testing errors as shown in Eq. (A6). The term $\Sigma _{D_{\mathrm{c}}, N_{\mathrm{c}}}$ represents the covariance between $D_{\mathrm{c}}, N_{\mathrm{c}}$, and similarly for all other covariances $\Sigma _{\mathrm{e}, N}$, $\Sigma _{D_{\mathrm{c}}, R_{\mathrm{c}}}$, $\Sigma _{R_{\mathrm{c}}, R_{\mathrm{u}}}$, $\Sigma _{R_{\mathrm{c}}, \gamma }$. Since variations in $D_{\mathrm{e}}$ arise from fluctuations in past-year baselines and not from current intrinsic uncertainty, we can neglect correlations between variations in $D_{\mathrm{e}}$ and uncertainty in $R_{\mathrm{c}}, R_{\mathrm{u}}$. The last two rows represent ${{{\mathcal {M}}}}$ expressed in two different ways, $\Gamma \equiv {\bar{D}}_{\mathrm{e}} + R_{\mathrm{c}} + R_{\mathrm{u}}$ and ${\bar{D}}_{\mathrm{e}} + R_{\mathrm{c}} + \gamma ({\bar{D}}_{\mathrm{e}} - D_\mathrm{c})$, respectively. Moreover, when using the SIR model to replace $D_{\mathrm{u}}$ and $R_{\mathrm{u}}$ with ${\bar{D}}_{\mathrm{e}}-D_{\mathrm{c}}\ge 0$, there is no uncertainty associated with $D_{\mathrm{u}}$ and $R_{\mathrm{u}}$ in a deterministic model. Thus, covariances cannot be defined except through the uncertainty in the parameter $\gamma = \gamma _\mathrm{u}/\mu _{\mathrm{u}}$

Mortality *Z*	Uncertainties	$\hbox {CV}^{2}\displaystyle ={\Sigma _{Z}^{2}\over Z^{2}}$
$\displaystyle \mathrm{CFR}={D_{\mathrm{c}}\over N_{\mathrm{c}}}$	$\Sigma _{D_{\mathrm{c}}}^{2}$, $\Sigma _{N_{\mathrm{c}}}^{2}$, $\Sigma _{D_{\mathrm{c}}, N_{\mathrm{c}}}$	$\displaystyle {\Sigma _{D_{\mathrm{c}}}^{2} \over D_{\mathrm{c}}^{2}}+ {\Sigma _{N_{\mathrm{c}}}^{2} \over N_{\mathrm{c}}^{2}} - {2\Sigma _{D_{\mathrm{c}}, N_{\mathrm{c}}} \over D_{\mathrm{c}}N_{\mathrm{c}}}$
$\displaystyle \overline{\mathrm{IFR}} \approx {{\bar{D}}_{\mathrm{e}} \over f\;N}$	$\Sigma _{\mathrm{e}}^{2}, \Sigma _{N}^{2}, \Sigma _{\mathrm{e},N}, \sigma _{f}^{2}$	$\displaystyle {{\sigma }_{f}^{2} \over {\hat{f}}^{2}}+ {\Sigma _{\mathrm{e}}^{2} \over {\bar{D}}_\mathrm{e}^{2}}+{\Sigma _{N}^{2} \over N^{2}}- {2\Sigma _{\mathrm{e}, N}\over {\bar{D}}_{\mathrm{e}}N}$
$\displaystyle M={D_{\mathrm{c}}\over D_{\mathrm{c}}+R_{\mathrm{c}}}$	$\Sigma _{D_{\mathrm{c}}}^{2}, \Sigma _{R_{\mathrm{c}}}^{2}, \Sigma _{D_{\mathrm{c}}, R_{\mathrm{c}}}$	$\displaystyle M^{2}\left( {R_{\mathrm{c}}\over D_{\mathrm{c}}}\right) ^{2} \left[ {\Sigma _{D_{\mathrm{c}}}^{2} \over D_{\mathrm{c}}^{2}}+ {\Sigma _{R_\mathrm{c}}^{2} \over R_{\mathrm{c}}^{2}} - {2\Sigma _{R_{\mathrm{c}}, D_\mathrm{c}}\over R_{\mathrm{c}}D_{\mathrm{c}}}\right]$
$\displaystyle \overline{{{{\mathcal {M}}}}} \approx {{\bar{D}}_{\mathrm{e}} \over {\bar{D}}_{\mathrm{e}} + R_{\mathrm{c}} +R_{\mathrm{u}}}$	$\Sigma _{\mathrm{e}}^{2}, \Sigma _{R_{\mathrm{c}}}^{2}, \Sigma _{R_{\mathrm{u}}}^{2}, \Sigma _{R_{\mathrm{c}}, R_{\mathrm{u}}}$	$\displaystyle (1-{{{\mathcal {M}}}})^{2}{\Sigma _{\mathrm{e}}^{2} \over {\bar{D}}_{\mathrm{e}}^{2}} +{\Sigma _{R_{\mathrm{c}}}^{2}\over \Gamma ^{2}} +{\Sigma _{R_{\mathrm{u}}}^{2}\over \Gamma ^{2}} -{2\Sigma _{R_{\mathrm{c}}, R_{\mathrm{u}}}\over \Gamma ^{2}}$
$\displaystyle \overline{{{{\mathcal {M}}}}} \approx {{\bar{D}}_{\mathrm{e}} \over {\bar{D}}_{\mathrm{e}} + R_{\mathrm{c}} + \gamma ({\bar{D}}_{\mathrm{e}}-D_{\mathrm{c}})}$	$\Sigma _{R_{\mathrm{c}}}^{2}, \Sigma _{\mathrm{e}}^{2}, \Sigma _{R_{\mathrm{c}}, \gamma }, \sigma _{\gamma }^{2}$	$\displaystyle (1-{{{\mathcal {M}}}})^{2} {\Sigma _{\mathrm{e}}^{2} \over {\bar{D}}_{\mathrm{e}}^{2}} + {\Sigma _{R_{\mathrm{c}}}^{2} \over \Gamma ^{2}} + {({\bar{D}}_{\mathrm{e}}-D_{\mathrm{c}})^{2}\sigma _{\gamma }^{2} \over \Gamma ^{2}} - {2({\bar{D}}_{\mathrm{e}}-D_{\mathrm{c}})\Sigma _{R_{\mathrm{c}}, \gamma }\over \Gamma ^{2}}$

We now use excess death data and the statistical and modeling procedures to estimate mortality measures $Z=$ $\mathrm{IFR}$, $\mathrm{CFR}$, *M*, ${{{\mathcal {M}}}}$ across different jurisdictions, including all US states and more than 75 countries.^1^ Accurate estimates of the confirmed infecteds $N_{\mathrm{c}}$ and the confirmed dead $D_{\mathrm{c}}$ are needed to evaluate the $\mathrm{CFR}$. Values for the parameters *Q*, $\mathrm{FPR}$, $\mathrm{FNR}$, and *b* are needed to estimate $N_{\mathrm{c}}+N_{\mathrm{u}} = f\;N$ in the denominator of the $\mathrm{IFR}$, while ${\bar{D}}_{\mathrm{e}}$ is needed to estimate the number of infection-caused deaths $D_{\mathrm{c}}+D_{\mathrm{u}}$ that appear in the numerator of the $\mathrm {IFR}$ and ${{{\mathcal {M}}}}$. Finally, since we evaluate the resolved mortality ${\mathcal {M}}$, through Eq. (8), estimates of $D_{\mathrm{e}}, D_{\mathrm{c}}, R_{\mathrm{c}}$, $\gamma$, and $\mathrm{FPR}$, $\mathrm{FNR}$ (to correct for testing inaccuracies in $D_{\mathrm{c}}$ and $R_{\mathrm{c}}$) are necessary. Whenever uncertainties are available or inferable from data, we also include them in our analyses.

Estimates of excess deaths and infected populations themselves suffer from uncertainty encoded in the variances $\Sigma _{\mathrm{e}}^{2}$ and $\sigma _{f}^{2}$. The uncertainty in *f* depends on the uncertainties arising from finite sampling sizes, uncertainty in bias *b* and uncertainty in test sensitivity and specificity, which are denoted $\sigma _{b}^{2}$, $\sigma _{\mathrm{I}}^{2}$, and $\sigma _{\mathrm{II}}^{2}$, respectively. We use $\Sigma ^2$ to denote variances in populations and $\sigma ^2$ to denote variances in intrinsic parameters; covariances with respect to any two variables *X*, *Y* are denoted as $\Sigma _{X,Y}$. Variances in the confirmed populations are denoted $\Sigma _{N_{\mathrm{c}}}^{2}$, $\Sigma _{R_{\mathrm{c}}}^{2}$, and $\Sigma _{D_{\mathrm{c}}}^{2}$ and also depend on uncertainties in testing parameters $\sigma _{\mathrm{I}}^{2}$ and $\sigma _{\mathrm{II}}^{2}$. The most general approach would be to define a probability distribution or likelihood for observing a value of the mortality index $Z\in [z, z+\text{ d }z]$. As outlined in the SI, these probabilities depend on the probability densities of the components of the mortalities, which in turn may depend on hyperparameters that define these probability densities. Here, we simply assume uncertainties that are propagated to the mortality indices through variances in the model parameters and hyperparameters [41]. The squared coefficients of variation of the mortalities are found by linearizing them about the mean values of the underlying components and are listed in Table 3.

**Fig. 4:**
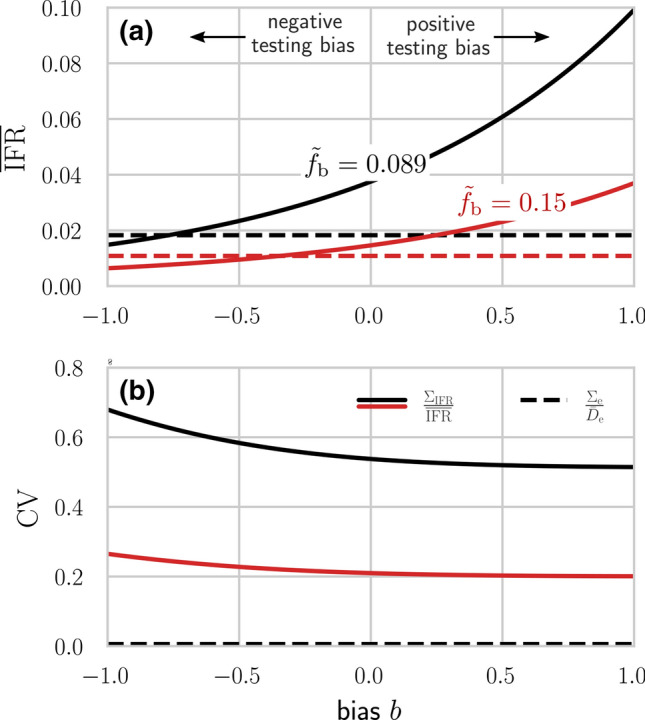
Different mortality measures across different regions. (a) The expected apparent (dashed lines) and true (solid lines) $\overline{\mathrm{IFR}}$s in the US from December 29, 2019 up until February 6, 2021, estimated using excess mortality data. We set ${\tilde{f}}_{\mathrm{b}}=0.089,0.15$ (black, red), $\mathrm{FPR}=0.05$, $\mathrm{FNR}=0.2$, and $N=330$ million. For the expected true $\overline{\mathrm{IFR}}$, we use ${\hat{f}}$ as defined in Eq. (6). Unbiased testing corresponds to setting $b=0$. For $b>0$ (positive testing bias), infected individuals are overrepresented in the sample population. Hence, the corrected $\overline{\mathrm {IFR}}$ is larger than the apparent $\overline{\mathrm {IFR}}$. If *b* is sufficiently small (negative testing bias), the expected true $\overline{\mathrm {IFR}}$ may be smaller than the expected apparent $\overline{\mathrm {IFR}}$. (b) The coefficient of variation of ${\bar{D}}_{\mathrm{e}}$ (dashed line) and $\overline{\mathrm{IFR}}$ (solid lines) with $\sigma _{\mathrm{I}}=0.02$, $\sigma _{\mathrm{II}}=0.05$, and $\sigma _{b}=0.2$ (see Table 3)

To illustrate the influence of different biases *b* on the $\mathrm {IFR}$ we use ${\hat{f}}$ from Eq. (6) in the corrected $\mathrm {IFR}\approx D_{\mathrm{e}}/({\hat{f}} N)$. We model RT–PCR-certified COVID-19 deaths [42] by setting the $\mathrm{FPR}=0.05$ [43] and the $\mathrm{FNR}=0.2$ [44, 45]. The observed, possibly biased, fraction of positive tests ${\tilde{f}}_\mathrm{b}$ can be directly obtained from corresponding empirical data.

As of February 6, 2021, the average of ${\tilde{f}}_{\mathrm{b}}$ over all tests and across all US states is about 9.3% [46]. The corresponding number of excess deaths is ${\bar{D}}_{\mathrm{{e}}}=536,\!617$ [29] and the US population is about $N\approx 330$ million [47]. To study the influence of variations in ${\tilde{f}}_{\mathrm{b}}$, in addition to ${\tilde{f}}_{\mathrm{b}}=0.089$, we also use a slightly larger ${\tilde{f}}_{\mathrm{b}}=0.15$ in our analysis. In Fig. 4 we use the ${\bar{D}}_{\mathrm{e}}$ from the US and show the expected apparent and corrected $\overline{\mathrm {IFR}}$s for two values of ${\tilde{f}}_\mathrm{b}$ [Fig. 4a] and the coefficient of variation $\mathrm {CV}_{\mathrm{IFR}}$ [Fig. 4b] as a function of the bias *b*. For unbiased testing [$b=0$ in Fig. 4a], the corrected $\mathrm {IFR}$ in the US is 3.6% assuming ${\tilde{f}}_\mathrm{b}=0.089$ and 1.4% assuming ${\tilde{f}}_{\mathrm{b}}=0.15$. If $b>0$, a higher proportion of the infected population is tested, hence, the expected apparent $\overline{\mathrm {IFR}} = {\bar{D}}_\mathrm{e}/({\tilde{f}}_{\mathrm{b}}N)$ is smaller than the true $\overline{\mathrm {IFR}}$, as can be seen by comparing the solid (corrected $\overline{\mathrm{IFR}}$) and the dashed (apparent $\overline{\mathrm{IFR}}$) lines in Fig. 4a. For testing biased towards the uninfected population ($b<0$), the corrected $\overline{\mathrm {IFR}}$ may be smaller than the apparent $\overline{\mathrm {IFR}}$. To illustrate how uncertainty in $\mathrm {FPR}$, $\mathrm {FNR}$, and *b* affect uncertainty in $\overline{\mathrm{IFR}}$, we evaluate $\hbox {CV}_{\mathrm{{IFR}}}$ as given in Table 3.

The first term in uncertainty $\sigma _{f}^{2}/{\hat{f}}^{2}$ given in Eq. (A6) in the SI is proportional to 1/*Q* and can be assumed to be negligibly small, given the large number *Q* of tests administered. The other terms in Eq. (A6) are evaluated by assuming $\sigma _{b}=0.2, \sigma _{\mathrm{I}}=0.02$, and $\sigma _\mathrm{II}=0.05$ and by keeping $\mathrm{FPR} = 0.05$ and $\mathrm{FNR} = 0.2$. Finally, we infer $\Sigma _{\mathrm{e}}$ from empirical data using Eq. (2), neglect correlations between $D_{\mathrm{e}}$ and *N*, and assume that the variation in *N* is negligible so that $\Sigma _{\mathrm{e},N} = \Sigma _{N} \approx 0$. Fig. 4b plots $\mathrm{CV}_{\overline{\mathrm {IFR}}}$ and $\mathrm{CV}_{D_{\mathrm{e}}}$ in the US as a function of the underlying bias *b*. The coefficient of variation $\mathrm{CV}_{D_{\mathrm{e}}}$ is about 1%, much smaller than $\mathrm{CV}_{\overline{\mathrm {IFR}}}$, and independent of *b*. For the values of *b* shown in Fig. 4b, $\mathrm{CV}_{\overline{\mathrm {IFR}}}$ is between 51–68% for ${\tilde{f}}_{\mathrm{b}}=0.089$ and between 20–27% for ${\tilde{f}}_\mathrm{b}=0.15$.

**Fig. 5:**
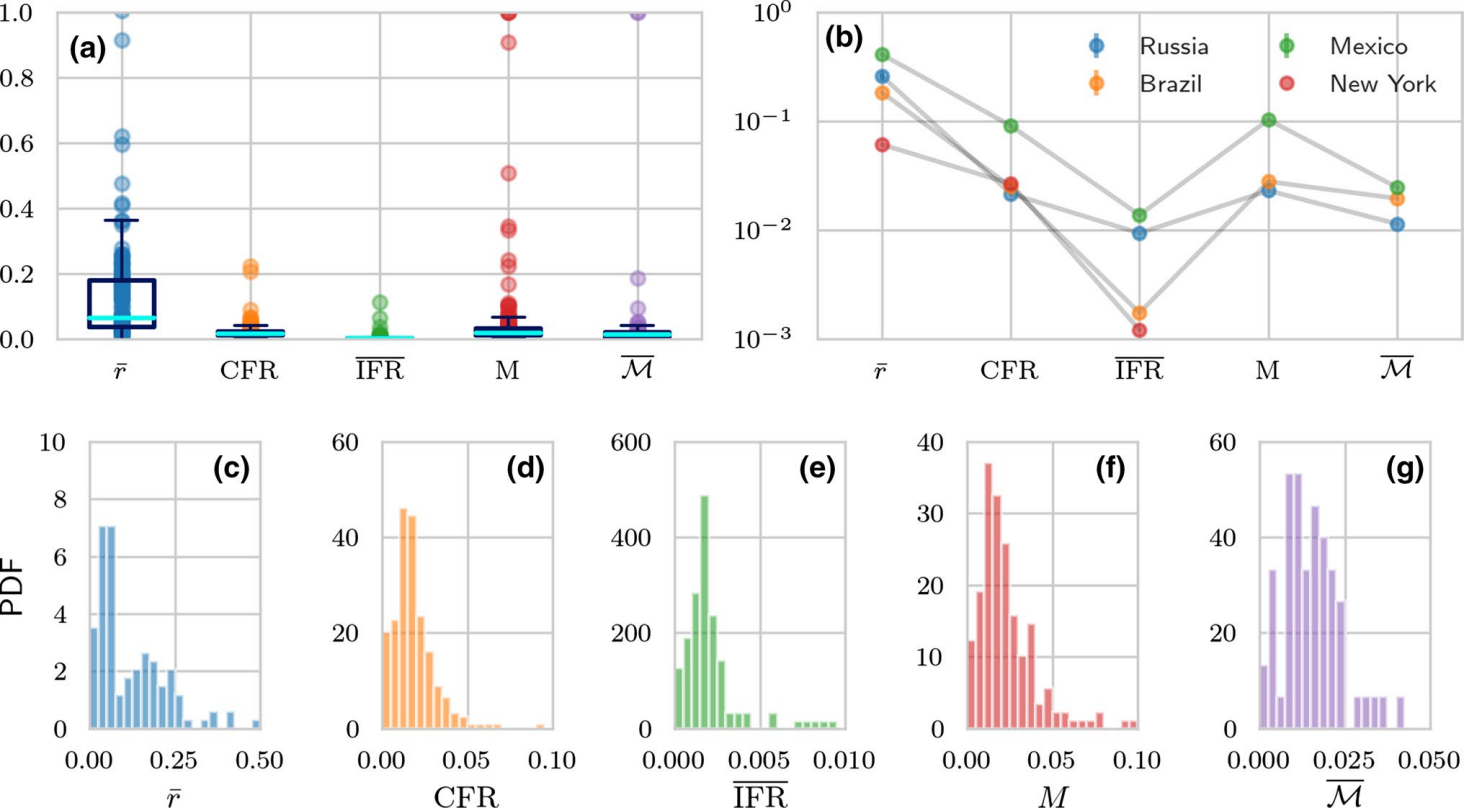
Mortality characteristics in different countries and states. (a) Running vales of relative excess deaths ${\bar{r}}$, the $\mathrm{CFR}$, the $\overline{\mathrm {IFR}}= p {\bar{D}}_{\mathrm {e}}/N_{\mathrm{c}}$ with $p=N_{\mathrm{c}}/(N_{\mathrm{c}}+N_\mathrm{u}) = 0.1$ [35], the confirmed resolved mortality *M*, and the expected true resolved mortality $\overline{{\mathcal {M}}}$ (using $\gamma =100$) are plotted for various jurisdictions. (b) Different mortality measures may be higher or lower in different jurisdictions, providing ambiguous characterization of disease severeness. (c–g) The probability density functions (PDFs) of the mortality measures shown in (a) and (b). Note that there are only very incomplete recovery data available for certain countries (*e.g.*, US and UK). For countries without recovery data, we could not determine *M* and $\overline{{\mathcal {M}}}$. The number of jurisdictions that we used in (a) and (c–g) are 136, 247, 127, 191, and 75 for the respective mortality measures (from left to right). All data from January 2020 onwards were included and last updated on March 30, 2021 [22, 29, 31, 40]

Next, we compared the mortality measures $\overline{\mathrm{IFR}}$, $\mathrm{CFR}$, *M*, $\overline{{{{\mathcal {M}}}}}$, and ${\bar{r}}$ listed in Table 1 across numerous jurisdictions. To determine the $\mathrm{CFR}$, we use the COVID-19 data of Refs. [22, 40]. For the expected apparent $\overline{\mathrm{IFR}}$, we use the representation $\overline{\mathrm{IFR}}= p{\bar{D}}_{\mathrm{e}}/N_{\mathrm{c}}$ discussed above. Although *p* may depend on the stage of the pandemic, typical estimates range from 4% [48] to 10% [35]. We set $p=0.1$ over the lifetime of the pandemic. We can also directly use $\overline{\mathrm{IFR}}= {\bar{D}}_{\mathrm{e}}/(f\;N)$, however estimating the corrected $\overline{\mathrm{IFR}}$ requires evaluating the bias *b*. In Fig. 5a, we show the running values (up until March 30, 2021) of the relative excess deaths ${\bar{r}}$, the $\mathrm{CFR}$, the apparent $\overline{\mathrm{IFR}}$, the confirmed resolved mortality *M*, and the true resolved mortality $\overline{{\mathcal {M}}}$ across all regions. In all cases we set $p = 0.1, \gamma =100$. As illustrated in Fig. 5b, some mortality measures suggest that COVID-induced fatalities are lower in certain countries compared to others, whereas other measures indicate the opposite. For example, the expected total resolved mortality $\overline{{\mathcal {M}}}$ for Brazil is larger than for Russia and Mexico, most likely due to the relatively low number of reported excess deaths as can be seen from Fig. 3a. On the other hand, Brazil’s values of $\mathrm {CFR}$, $\overline{\mathrm {IFR}}$, and *M* are substantially smaller than those of Mexico [see Fig. 5b].

The distributions of all measures *Z* and relative excess deaths *r* across jurisdictions are shown in Fig. 5c–g and encode the global uncertainty of these indices. We also calculate the corresponding mean values across jurisdictions, and use the empirical cumulative distribution functions to determine confidence intervals. The average of the mortality indices values across all jurisdictions are $\langle {\bar{r}}\rangle =0.121$ (95% CI: 0.000–0.594), $\langle \mathrm {CFR}\rangle =0.020$ (95% CI: 0.000–0.050), $\langle \overline{\mathrm {IFR}}\rangle =0.004$ (95% CI: 0.00–0.02), $\langle M\rangle = 0.039$ (95% CI: 0.000–0.242), and $\langle \overline{{\mathcal {M}}}\rangle = 0.021$ (95% CI: 0.000–0.094). Here, the CI measures the variation across jurisdictions. To calculate $\langle M\rangle$ and $\langle \overline{{\mathcal {M}}}\rangle$, we excluded countries with incomplete recovery data. The distributions plotted in Fig. 5c–g can be used to inform our analyses of uncertainty or heterogeneity as summarized in Table 3. For example, the overall variance $\Sigma _{Z}^{2}$ can be determined by fitting the corresponding empirical *Z* distribution shown in Fig. 5c–g. Table 3 displays how the related $\mathrm {CV}_{Z}^{2}$ can be decomposed into separate terms, each arising from the variances associated to the components in the definition of *Z*. For concreteness, from Fig. 5e we obtain $\mathrm{CV}^2_{\overline{\mathrm{IFR}}} =\Sigma _{\overline{\mathrm{IFR}}}^2/ \langle \overline{\mathrm {IFR}}\rangle ^{2} \approx 8.05$ which allows us to place an upper bound on $\sigma _{b}^{2}$ using Eq. (A6), the results of Table 3, and

9 $$\begin{aligned} \sigma _{b}^{2} < \frac{({\tilde{f}}_{\mathrm{b}}-\mathrm{FPR})^{2}}{{\hat{f}}^{2}(1-{\hat{f}})^{2}}\mathrm{CV}^{2}_{\overline{\mathrm{IFR}}} \approx \frac{({\tilde{f}}_{\mathrm{b}}-\mathrm{FPR})^{2}}{{\hat{f}}^{2}(1-{\hat{f}})^{2}} 8.05 \end{aligned}$$

or on $\sigma _{\mathrm{I}}^{2}$ using $(1-{\hat{f}})^{2}\sigma _{\mathrm{I}}^{2} < ({\tilde{f}}_{\mathrm{b}}-\mathrm{FPR})^{2}\mathrm{CV}^{2}_{\overline{\mathrm{IFR}}}$.

**Fig. 6:**
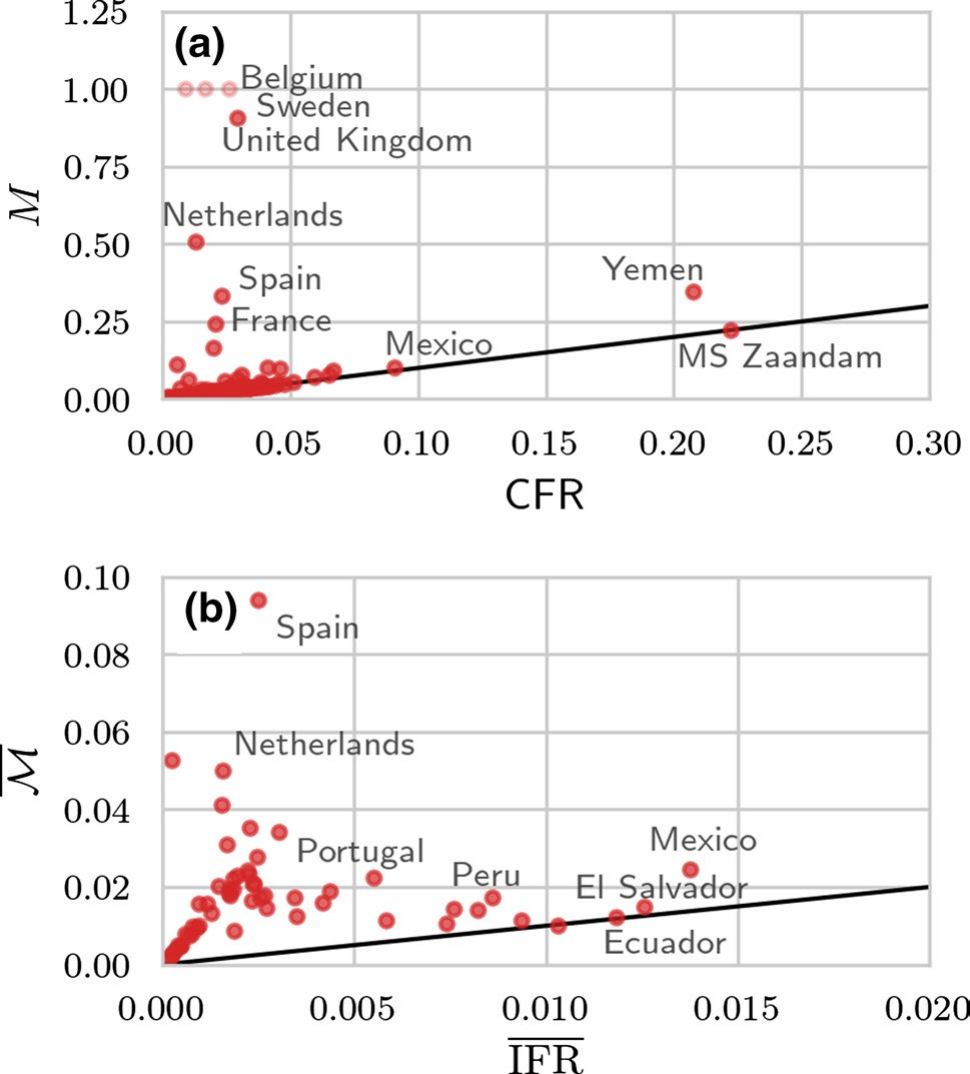
Different mortality measures across different regions. We show the values of *M* and $\mathrm {CFR}$ (a) and $\overline{{\mathcal {M}}}$ (using $\gamma =100$) and $\overline{\mathrm {IFR}}= p {\bar{D}}_{\mathrm {e}}/N_{\mathrm{c}}$ with $p=N_{\mathrm{c}}/(N_{\mathrm{c}}+N_{\mathrm{u}}) = 0.1$ [35] (b) for different jurisdictions. The black solid lines have slope 1. If jurisdictions do not report the number of recovered individuals, $R_{\mathrm{c}} = 0$ and $M =1$ [light red circles in (a)]. In jurisdictions for which the data indicate ${\bar{D}}_{\mathrm{e}} < D_\mathrm{c}$, we set $\gamma ({\bar{D}}_{\mathrm{e}} - D_{\mathrm{c}}) = 0$ in the denominator of $\overline{{{{\mathcal {M}}}}}$ which prevents it from becoming negative as long as ${\bar{D}}_{\mathrm{e}} \ge 0$. All data were counted from January 2020 onwards and last updated on March 30, 2021 [22, 29, 31, 40]

Finally, to provide more insight into the correlations between different mortality measures, we plot *M* against $\mathrm {CFR}$ and ${\mathcal {M}}$ against $\mathrm {IFR}$ in Fig. 6. For most regions, we observe similar values of *M* and $\mathrm {CFR}$ in Fig. 6a. Although we expect $M \rightarrow \mathrm {CFR}$ and ${\mathcal {M}} \rightarrow \mathrm {IFR}$ towards the end of an epidemic, in some jurisdictions such as the UK, the Netherlands, and Sweden, $M \gg \mathrm{CFR}$ due to unreported or incomplete reporting of recovered cases. About 30% of the regions that we show in Fig. 6b have an $\overline{\mathrm {IFR}}$ that is approximately equal to $\overline{{\mathcal {M}}}$. Again, for regions such as Sweden and the Netherlands, $\overline{{\mathcal {M}}}$ is substantially larger than $\overline{\mathrm {IFR}}$ because of incomplete reporting of recovered cases.

## Discussion

In this paper, we review running COVID mortality metrics starting from the onset of the pandemic through December 2020, or later dates where data were available. In the first few weeks of the initial COVID-19 outbreak in March and April 2020 in the US, the reported death numbers captured only about two thirds of the total excess deaths [17]. This mismatch may have arisen from reporting delays, attribution of COVID-19 related deaths to other respiratory illnesses, and secondary pandemic mortality resulting from delays in necessary treatment and reduced access to health care [17]. We also observe that the number of excess deaths in the Fall months of 2020 have been significantly higher than the corresponding reported COVID-19 deaths in many US states and countries. The weekly numbers of deaths in regions with a high COVID-19 prevalence were up to 8 times higher than in previous years. Among the countries that were analyzed in this study, the ten countries with the largest numbers of excess deaths since the beginning of the COVID-19 outbreak (all numbers per 100,000) are Peru (358), Russia (291), Lithuania (270), North Macedonia (270), Bulgaria (266), Mexico (241), Ecuador (237), Serbia (233), South Africa (217), and Poland (207). The ten countries with the lowest numbers of excess deaths since the beginning of the COVID-19 outbreak are Mongolia (-20), Malaysia (-16), Costa Rica (-15), Australia (-14), Japan (-11), Philippines (-11), New Zealand (-8), Singapore (-6), Mauritius (-6), and Georgia (-4) [29]. From this point of view, as of March, 2021, these countries have not experienced statistically significant higher mortality due to COVID-19.

The proposed use of excess deaths in standard mortality measures may provide a more meaningful estimate of infection-caused deaths, while errors in the estimates of the fraction of infected individuals in a population from testing can be corrected by estimating the testing bias, specificity, and sensitivity.

Underlying our use of excess deaths ${\bar{D}}_{\mathrm{e}}$ for evaluating the disease $\overline{\mathrm{IFR}}$ and mortality $\overline{{{{\mathcal {M}}}}}$ is the assumption that behavioral changes during the pandemic (social distancing, mask-wearing, lockdowns) had no appreciable effect on death. For example, the mean traffic deaths per month in Spain between 2011-2016 is about 174 persons [49], so any pandemic-related changes to traffic volumes would have little impact considering the much larger number of COVID-19 deaths. However, other deaths due to, *e.g.*, increased suicides and deferred medical treatment may contribute significantly to ${\bar{D}}_{\mathrm{e}}$. One could sharpen estimates of the true COVID-19 deaths by systematically analyzing the statistics of deaths from all reported causes using a standard protocol such as ICD-10 [50]. More research is necessary to disentangle the excess deaths that are directly caused by SARS-CoV-2 infections from those that result from postponed medical treatment [17], increased suicide rates [51], and other indirect factors contributing to an increase in excess mortality. Even if the numbers of excess deaths were accurately reported and known to be caused by a given disease, inferring the corresponding number of unreported cases (*e.g.*, asymptomatic infections), which appears in the definition of the $\mathrm {IFR}$ and ${\mathcal {M}}$ (see Table 1), is challenging and only possible if additional models and assumptions are introduced.

Different mortality measures are sensitive to different sources of uncertainty. Under the assumption that all excess deaths are caused by a given infectious disease (*e.g.*, COVID-19), the underlying error in the determined number of excess deaths can be estimated using historical death statistics from the same jurisdiction. Uncertainties in mortality measures can also be decomposed into the uncertainties of their component quantities, including that of the positive fraction *f* that in turn depend on uncertainties in the testing parameters. While we have considered only the average or last values of ${\tilde{f}}_{\mathrm{b}}$, our framework can be straightforwardly extended and dynamically applied across successive time windows, using *e.g.*, Bayesian or Kalman filtering approaches.

As for all epidemic forecasting and surveillance, our methodology depends on the quality of excess death and COVID-19 case data and knowledge of testing parameters. For many countries, the lack of binding international reporting guidelines, testing limitations, and possible data tampering [52] complicates the application of our framework. A striking example of variability is the large discrepancy between mean excess deaths ${\bar{D}}_{\mathrm{e}}$ and confirmed deaths $D_{\mathrm{c}}$ across many jurisdictions which render mortalities that rely on $D_{\mathrm{c}}$ suspect.

Finally, we have not partitioned the excess deaths or mortalities into subpopulations in age or other attributes such as sex, co-morbidities, occupation, etc. By expanding our testing and modeling approaches on stratified data, one can also straightforwardly infer stratified mortality measures *Z*, providing additional informative indices for comparison.

## Conclusions

Based on the data presented in Figs. 5 and 6, we conclude that the mortality measures ${\bar{r}}$, $\mathrm {CFR}$, $\mathrm {IFR}$, *M*, and ${\mathcal {M}}$ defined in Table 1 may provide different characterizations of disease severity across jurisdictions due to differences in testing bias and reporting protocols. The propagation of uncertainty and coefficients of variation that we summarize in Table 3 can help quantify and compare errors arising in different mortality measures, thus informing our understanding of the actual death toll of COVID-19. Depending on the stage of an outbreak and the currently available disease monitoring data, certain mortality measures are preferable to others. If the number of recovered individuals is being monitored, the resolved mortalities *M* and ${\mathcal {M}}$ should be preferred over $\mathrm {CFR}$ and $\mathrm {IFR}$, since the latter suffer from errors associated with the time-lag between infection and resolution [12]. For estimating $\mathrm {IFR}$ and ${\mathcal {M}}$, we propose using excess death data and an epidemic model. In situations in which case numbers cannot be estimated accurately, the relative excess deaths *r* provides a complementary measure to monitor disease severity. Our analyses of different mortality measures reveal that

- The $\mathrm{CFR}$ and *M* are defined directly from confirmed deaths $D_{\mathrm{c}}$ and suffers from variability in its reporting. Moreover, the $\mathrm {CFR}$ does not consider resolved cases and is expected to evolve during an epidemic. Although *M* includes resolved cases, it also requires confirmed recovered cases $R_\mathrm{c}$, adding to its variability across jurisdictions. Testing errors affect both $D_{\mathrm{c}}$ and $R_{\mathrm{c}}$, but if the $\mathrm{FNR}$ and $\mathrm{FPR}$ are known, they can be controlled using Eq. (A3) given in the SI.
- The $\mathrm{IFR}$ requires knowledge of the true cumulative number of disease-caused deaths as well as the true number of infected individuals (recovered or not) in a population. We show how these can be estimated from excess deaths and testing, respectively. Thus, the $\mathrm{IFR}$ will be sensitive to the inferred excess deaths and from the testing (particularly from the bias in the testing). Across all countries analyzed in this study, we found a mean $\mathrm {IFR}$ of about 0.44% (95% CI: 0.0–2.0%), which is similar to the previously reported values between 0.1 and 1.5% [35–37].
- In order to estimate the true resolved mortality ${{{\mathcal {M}}}}$, an additional relationship is required to estimate the unconfirmed recovered population $R_{\mathrm{u}}$. In this paper, we propose a simple SIR-type model in order to relate $R_{\mathrm{u}}$ to measured excess and confirmed deaths through the ratio of the recovery rate to the death rate. The variability in reporting $D_{\mathrm{c}}$ across different jurisdictions generates uncertainty in ${{{\mathcal {M}}}}$ and reduces its reliability when compared across jurisdictions.
- The mortality measures that can most reliably be compared across jurisdictions should not depend on reported data which are subject to different protocols, errors, and manipulation/intentional omission. Thus, the per capital excess deaths and relative excess deaths *r* (see last column of Table 1) should provide the most consistent comparisons of disease mortality across jurisdictions, provided total deaths are accurately tabulated. However, they are the least informative in terms of disease severity and individual risk, for which *M* and ${{{\mathcal {M}}}}$ are better.
- Uncertainty in all mortalities *Z* can be decomposed into the uncertainties in component quantities such as the excess death or testing bias. We can use global data to estimate the means and variances in *Z*, allowing us to put bounds on the variances of the component quantities and/or parameters.

Parts of our framework can be readily integrated into or combined with mortality surveillance platforms such as the European Mortality Monitor (EURO MOMO) project [30] and the Mortality Surveillance System of the National Center for Health Statistics [23] to assess disease burden in terms of different mortality measures and their associated uncertainty.

## Supplementary Information

Below is the link to the electronic supplementary material.

Supplementary material 1 (pdf 1248 KB)

## Data Availability

All datasets used in this study are available from Refs. [23–27, 29, 31]. The CDC excess death numbers and CIs are based on an overdispersed Poisson generalized linear model [31]. For the excess death data of Ref. [29], we determined CIs using the linear regression model class in R. The source codes used in our analyses are publicly available at [19].

## Footnotes

1 We provide an online dashboard that shows the real-time evolution of $\mathrm{CFR}$ and *M* at https://submit.epidemicdatathon.com/#/dashboard
