# Supplementary material for: Using excess deaths and testing statistics to determine COVID-19 mortalities
Source: Eur J Epidemiol. 2021 May 17;36(5):545–58. doi: 10.1007/s10654-021-00748-2 (PMC8127858; doi:10.1007/s10654-021-00748-2)
Supplement: Supplementary file 1 — Supplementary material 1 (pdf 1248 KB) [file 10654_2021_748_MOESM1_ESM.pdf]

## SUPPLEMENTARY INFORMATION

### Examples of excess death data

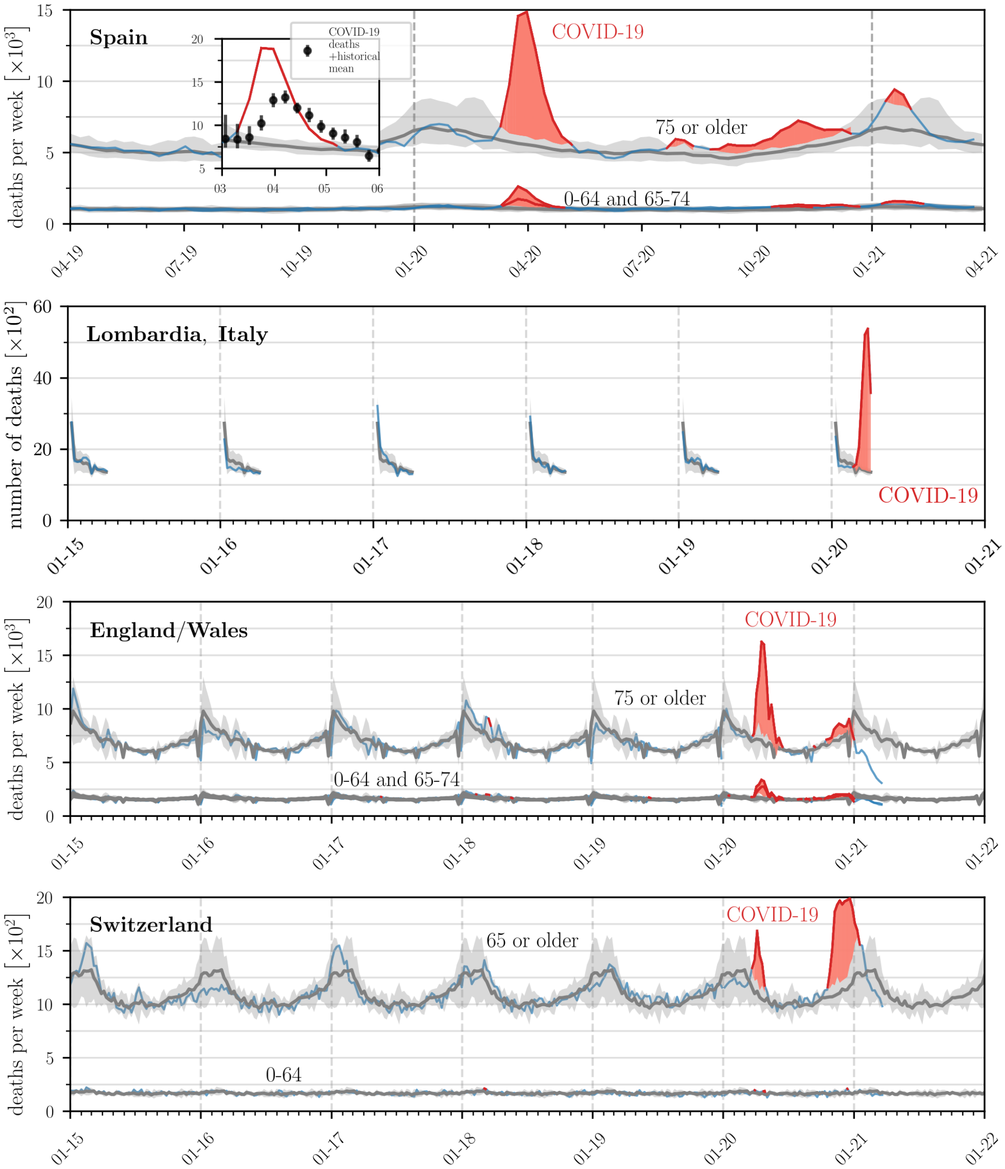


FIG. A1. **Mortality evolution in different countries**. The evolution of weekly deaths in Spain, England/Wales, and Switzerland for different age classes (where available). Solid grey curves and shaded regions represent the historical mean numbers of deaths and corresponding confidence intervals. Solid blue curves indicate weekly deaths and weekly deaths that lie outside the confidence intervals are indicated by solid red lines. For England/Wales and Switzerland, weekly means and 95% confidence intervals are based on data from 2015–2019. In the case of Spain, we show the reported COVID-19 deaths across all age classes [39] in the inset and use the 99% confidence intervals that are directly provided in the corresponding data [28]. The red shaded regions represent statistically significant cumulative excess deaths $D_e$. The data are derived from Refs. [23–27].

We tally weekly deaths according to Eq. (1) for each week $i$ starting from the first week of 2020, and cumulative excess deaths as in Eq. (2) adding all weekly contributions throughout 2020 and 2021. Note that some governmental agencies tabulate weekly deaths starting on the Sunday closest to January 1 2020 (December 29 2019, such as the United States), others instead use January 1 2020 as the first day of the week (such as Germany). A detailed list of how each country bins weekly deaths is included in Ref. [29]. The final week $k$ up to which the cumulative count is taken depends on data availability, since some countries have larger reporting delays than others. In the majority

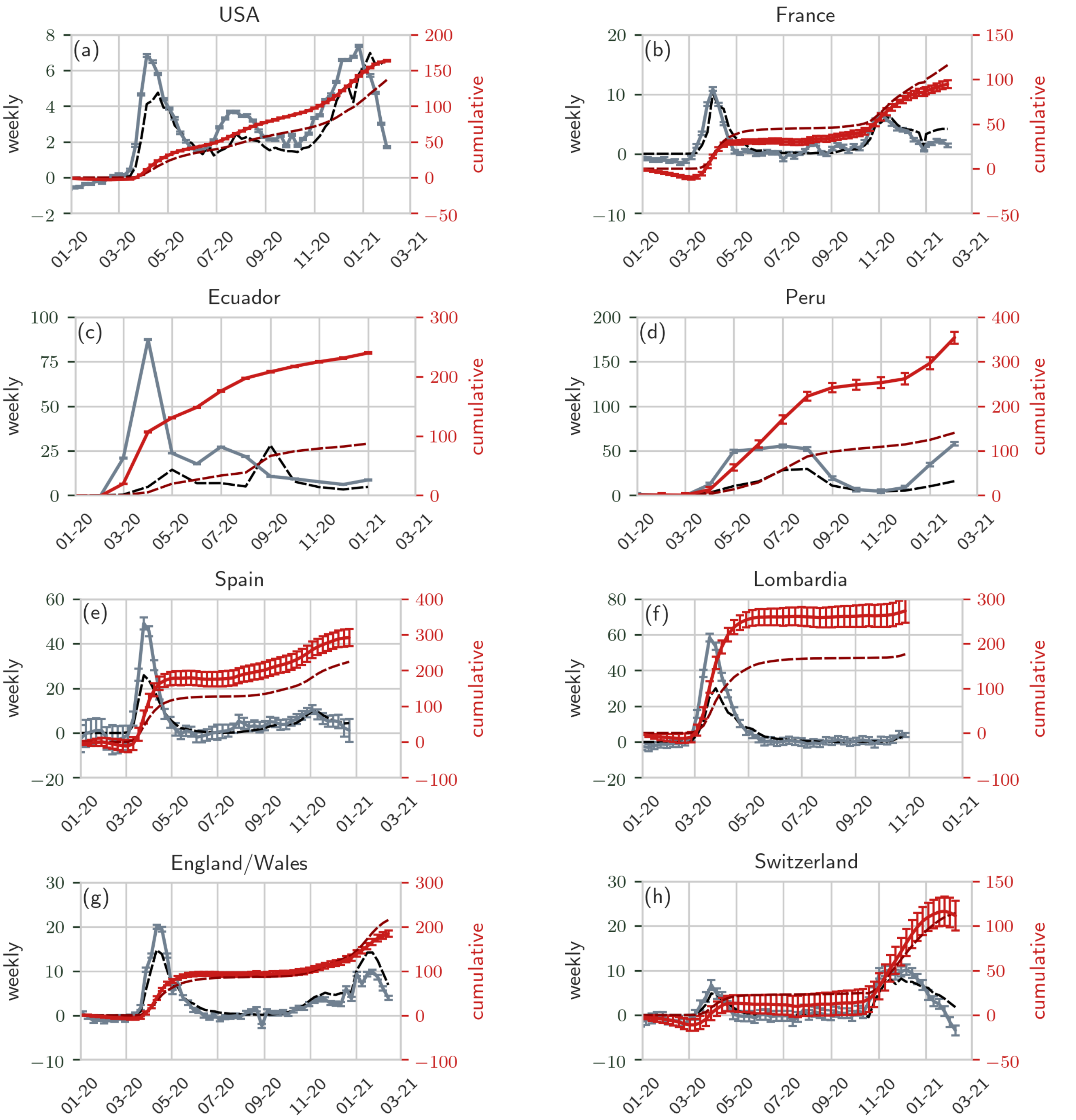


FIG. A2. **Weekly and cumulative death rates in different countries and regions**. We compare the evolution of confirmed weekly deaths $d_{\mathrm{c}}^{(0)}(i)$ (dashed black curves) and cumulative deaths $D_{\mathrm{c}}(k)$ (dashed dark red curves) with weekly excess deaths $\bar{d}_{\mathrm{e}}(i)$ (solid grey curves) and cumulative excess deaths $\bar{D}_{\mathrm{e}}(k)$ (solid red curves). The deaths are plotted in units of per 100,000 in different countries and regions. The data are derived from Ref. [29] and the error bars for the excess deaths are derived from Eqs. (1) and (2). For Spain, we used the 99% confidence intervals that are directly provided in the corresponding data [28] to approximate the 95% confidence intervals. Typically, we find $\bar{D}_{\mathrm{e}}(k) > D_{\mathrm{c}}(k)$.

of cases, $k$ is beyond December 2020. Quantities are calculated from data that include deaths from typically $J = 5$ previous years [29]. Figure A1 shows the evolution of weekly deaths over the past 2–6 years in Spain, Lombardia, England/Wales, and Switzerland. For these and other jurisdictions, we plot the weekly confirmed deaths $d_{\mathrm{c}}^{(0)}(i)$, the cumulative deaths $D_{\mathrm{c}}(k) = \sum_{i=1}^{k} d_{\mathrm{c}}^{(0)}(i)$, and the mean weekly and cumulative excess deaths $\bar{d}_{\mathrm{e}}(i)$ for 2020 as

available from data in Fig. A2. We also show $\bar{D}_{\mathrm{e}}(k)$ per 100,000 persons from the start of 2020 through March 1, 2021. The corresponding error bars in Fig. A2 indicate 95% confidence intervals defined by $\bar{d}_{\mathrm{e}}(i) \pm 1.96\,\sigma_{\mathrm{e}}(i)$ and $\bar{D}_{\mathrm{e}}(k) \pm 1.96\,\Sigma_{\mathrm{e}}(k)$ in Eqs. (1) and (2), respectively. For Spain, we used the 99% confidence intervals that are directly provided in the corresponding data [28] to approximate the 95% confidence intervals. Excess death statistics evolve differently across different countries and regions. For example, in France excess deaths were negative until the end of March 2020, quickly increasing in April 2020. In Ecuador and Peru, the number of excess deaths is more than 2.5 times larger than the corresponding number of confirmed COVID-19 deaths.

**Statistical testing model**

Given biases in sampling and testing errors, it is important to use a statistical testing model that takes them into account when estimating the fraction $f$ of a population $N$ that are infected. Testing biases arises, for example, if symptomatic individuals are more likely to seek testing. Thus, the fraction $f_{\mathrm{b}}$ of selected individuals who test positive may be different from $f$, the true fraction of infecteds. The relationship between $f$ and $f_{\mathrm{b}}$ is defined in Eq. (3). To derive Eq. 4, we start with the probability that $Q^+$ positive results arise from the $Q \geq Q^+$ error-free tests, given by

$$P_{\mathrm{true}}(Q^+|Q, f_{\mathrm{b}}) = \binom{Q}{Q^+} f_{\mathrm{b}}^{Q^+}(1-f_{\mathrm{b}})^{Q-Q^+}. \tag{A1}$$

This result is derived under the assumption that once individuals are tested, they are "replaced" in the population and can be tested again. The analogous distribution $P_{\mathrm{true}}(Q^+|Q, f_{\mathrm{b}})$ for testing "without replacement" can be straightforwardly derived and yields results similar to those in Eq. (A1).

Equation (A1) also assumes flawless testing. Tests with Type I (false positives) and Type II (false negatives) errors may wrongly catalog uninfected individuals as infected (with rate FPR) while missing some infected individuals (with rate FNR). For serological COVID-19 tests, such as antibody tests, the estimated percentages of false positives and false negatives are typically low, with FPR $\approx 0.03-0.07$ and FNR $\approx 0.1$ [43, 53, 54]. For RT-PCR tests, the FNRs depend strongly on the actual assay method [55, 56] and typically lie between 0.1 and 0.3 [44, 45] but might be as high as FNR $\approx 0.68$ if throat swabs are used [43, 45]. FNRs can also vary significantly depending on how long after initial infection the test is administered [57]. A systematic review conducted worldwide found FNR $\approx 0.54$ at initial testing [58], underlying the need for retesting. Reported percentages of false positives in RT-PCR tests are about FPR $\approx 0.05$ [43]. A large meta-analysis of serological tests estimates FPR $\approx 0.02$ and FNR $\approx 0.02-0.16$ [57]. These testing errors can lead to inaccurate estimates of disease prevalence; uncertainty in FPR, FNR will thus lead to uncertainty in the estimate of prevalence.

As illustrated through Fig. 2, errors in testing may result in the recorded number $\tilde{Q}^+$ of positive tests to be different from the $Q^+$ that would be obtained under perfect testing. The probability that $\tilde{Q}^+$ positive tests are returned due to testing errors can be described in terms of $Q^+$, FPR, and FNR and the corresponding probability distribution $P_{\mathrm{err}}(\tilde{Q}^+|Q^+, \mathrm{FPR}, \mathrm{FNR})$ is given by

$$P_{\mathrm{err}}(\tilde{Q}^+|Q, \mathrm{FPR}, \mathrm{FNR}) = \sum_{p_+=0}^{\tilde{Q}^+} \binom{Q^+}{p_+}(1-\mathrm{FNR})^{p_+}(\mathrm{FNR})^{Q^+-p_+}\binom{Q^-}{q_+}(\mathrm{FPR})^{q_+}(1-\mathrm{FPR})^{Q^--q_+}. \tag{A2}$$

where $Q^- \equiv Q - Q^+$ and the summation indices are related by $q_+ \equiv \tilde{Q}^+ - p_+$. By convolving $P_{\mathrm{err}}(\tilde{Q}^+|Q^+, \mathrm{FPR}, \mathrm{FNR})$ with $P_{\mathrm{true}}(Q^+|Q, f_{\mathrm{b}}(f, b))$ we derive the overall likelihood distribution for the measured number $\tilde{Q}^+$ of true *and* false positives given a set of specified parameters $\theta_{\mathrm{T}} = \{Q, b, \mathrm{FPR}, \mathrm{FNR}\}$ describing the population and testing

$$P(\tilde{Q}^+|f, \theta_{\mathrm{T}}) = \sum_{Q^+=0}^{Q} P_{\mathrm{err}}(\tilde{Q}^+|Q^+, \mathrm{FPR}, \mathrm{FNR}) P_{\mathrm{true}}(Q^+|Q, f_{\mathrm{b}}(f, b)). \tag{A3}$$

Using Gaussian approximations (according to the de Moivre–Laplace theorem) for $P_{\mathrm{true}}(Q^+|Q, f_b)$ and $P_{\mathrm{err}}(\tilde{Q}^+|Q^+, \mathrm{FPR}, \mathrm{FNR})$, we can express $P(\tilde{f}_{\mathrm{b}}|f, \theta_{\mathrm{T}})$ as a normal distribution over the observed positive fraction $\tilde{f}_{\mathrm{b}} := \tilde{Q}^+/Q$ [Eqs. (4) and (5)]. These approximations are valid if (i) the number of positive and apparently positive tests, $Q^+$ and $\tilde{Q}^+$ (and hence the total number of tests $Q$) are sufficiently large, and (ii), the quantities $f$, FNR, and FPR are not too close to 0 or 1.

Using Bayes' rule, we can then formally define the likelihood of $f$ given a measured value $\tilde{f}_{\mathrm{b}}$,

$$P(f|\tilde{f}_{\rm b}, \theta_{\rm T} \equiv \{b, {\rm FPR}, {\rm FNR}\}) = \frac{P(\tilde{f}_{\rm b}|f, \theta_{\rm T})P_0(f)}{\int P(\tilde{f}_{\rm b}|f, \theta_{\rm T})P_0(f){\rm d}f}, \tag{A4}$$

where $P(f|\tilde{f}_{\rm b}, \theta_{\rm T})$ is given by Eq. (4) and $P_0(f)$ is a prior on $f$, such as $\mathrm{U}(0, f_{\rm max})$. Eq. (A4) then yields the posterior $P(f|\tilde{f}_{\rm b}, \theta_{\rm T})$ when the testing parameters $\theta_{\rm T} \equiv \{b, {\rm FPR}, {\rm FNR}\}$ are independently and accurately specified. However, uncertainty in $\theta_{\rm T}$ can be encoded in distributions $P_0(\theta_{\rm T} = \{b, {\rm FPR}, {\rm FNR}\}|\alpha_{\rm T})$ which can be used as a weight for the posterior distribution over $f$. The hyperparameters $\alpha_{\rm T}$ that define $P_0(\theta_{\rm T}|\alpha_{\rm T})$ may include the expected values $\{\bar{b}, \overline{\rm FPR}, \overline{\rm FNR}\}$ and standard deviations $\{\sigma_b, \sigma_{\rm I}, \sigma_{\rm II}\}$ obtained from independent measurements/estimates.

A formal statistical analysis of the mortalities can be carried out by be finding the probability density of measuring a value of $Z \in [z, z + {\rm d}z]$, $P_Z(z|\alpha) = \int P_Z(z|\theta)P(\theta|\alpha){\rm d}\theta$, where $P_Z(z|\theta)$ defines the density of the value of $Z$ at $z$ given $\theta$ includes all variables used to determine $Z$. The associated hyperparameters $\alpha$ define the distribution over all component variables $\theta$. For example, if $Z = M$, then $\theta = \{D_{\rm c}, R_{\rm c}\}$ plus any other parameters associated with the measurement of $D_{\rm c}$ and $R_{\rm c}$. On the other hand, if $Z \equiv {\rm IFR} = D_{\rm e}/(fN)$, we also need to evaluate the statistics of $D_{\rm e}$ and $N$. Thus, the full set of parameters are $\theta = \{D_{\rm e}, N, \theta_{\rm T}\}$ and the hyperparameters $\alpha$ could be $\{(\bar{D}_{\rm e}, \Sigma_{\rm e}), (\bar{N}, \Sigma_N), (\bar{b}, \overline{\rm FPR}, \overline{\rm FNR}, \sigma_b, \sigma_{\rm I}, \sigma_{\rm II})\}$, the mean and standard deviations of the excess deaths, total population, and testing parameters, respectively (assuming $Q$ tests are known to be given). To be explicit, we consider the simpler case where $N, Q, b$, FPR, and FNR are precisely known (incorporating their uncertainties is straightforward but mathematically cumbersome). The probability density for observing ${\rm IFR} \in [z, z + {\rm d}z]$, in terms of the remaining component variables $Y = \{D_{\rm e}, f\} \subseteq \theta$ can be decomposed according to

$$\begin{aligned} P({\rm IFR} = z) &= \int P_{\rm IFR}(z|y)P_Y(y|\tilde{f}_{\rm b}){\rm d}y \\ &= \iint \delta\left(z - \frac{D_{\rm e}}{fN}\right) P_{\rm e}(D_{\rm e}|\alpha_{\rm e})P(f|\tilde{f}_{\rm b})\,{\rm d}D_{\rm e}{\rm d}f \\ &= \int P_{\rm e}(fNz|\alpha_{\rm e})P(f|\tilde{f}_{\rm b})fN\,{\rm d}f \end{aligned} \tag{A5}$$

where in the second equality we have used the definition of ${\rm IFR} = D_{\rm e}/(fN)$ through the Dirac $\delta$-function for $P_{\rm IFR}$, and approximated $P_Y(y|\tilde{f}_{\rm b})$ with the product $P_{\rm e}(D_{\rm e}|\alpha_{\rm e})P(f|\tilde{f}_{\rm b})$.[2] Here, the distribution over excess deaths $P_{\rm e}$ is defined by, for example, $\alpha_{\rm e} = \{\bar{D}_{\rm e}, \Sigma_{\rm e}\}$. The third equality uses a change of variables and involves the posterior distribution $P(f|\tilde{f}_{\rm b})$ which is a simple case of Eq. (A4) when $b$, FPR, and FNR are perfectly known. One can use a log-normal or gamma distribution for $P_{\rm e}$ with mean and variance defined using Eqs. (2) and a uniform prior $\mathrm{U}(0, f_{\rm max})$ in Eq. (A4) to find the posterior density $P(f|\tilde{f}_{\rm b})$.[3] Eq. (A5) can then be explicitly evaluated to find the probability density for the IFR given $\tilde{f}_{\rm b}$. Similar convolutions and change-of-variable calculations can be performed to derive densities for all other indices $Z$.

A simpler way to approximate uncertainty in the infected fraction $f$ is to assume a Gaussian approximation for all distributions and propagate the uncertainty in testing parameters. The squared coefficient of variation ${\rm CV}_f^2$ is then decomposed into the parameter variances according to

$$\frac{\sigma_f^2}{\hat{f}^2} \approx \frac{(1-(1-e^b)\hat{f})^2}{X^2 Q}\tilde{f}_{\rm b}(1-\tilde{f}_{\rm b}) + \frac{(1-\hat{f})^2}{X^2}\sigma_{\rm I}^2 + \frac{e^{2b}\hat{f}^2}{X^2}\sigma_{\rm II}^2 + \frac{\hat{f}^2(1-\hat{f})^2}{X^2}\sigma_b^2, \tag{A6}$$

where $X \equiv \tilde{f}_{\rm b} - {\rm FPR}$. The values of $b$, FPR, FNR above are mean or maximum likelihood estimates of the bias and testing errors, and $\sigma_b^2$, $\sigma_{\rm I}^2$, and $\sigma_{\rm II}^2$ are their associated uncertainties. Our result for $\sigma_f^2$ in Eq. (A6) assumes $\{b, {\rm FPR}, {\rm FNR}\}$ are uncorrelated. Since $Q \gg 1$ is typically large, we expect the first contribution to the uncertainty, arising from stochasticity in the sampling and proportional to $\tilde{f}_{\rm b}(1-\tilde{f}_{\rm b})/Q$ to be negligible. Uncertainties in other quantities will ultimately contribute to uncertainty in the mortalities $Z$, as listed in Table III.

---

[2] Since the distribution over $D_{\rm e}$ is constructed from data from previous years, and $\tilde{f}_{\rm b} := \tilde{Q}^+/Q$ is measured this year, we assume that they are approximately independent variables. We also assumed that the total population $N$ is perfectly known.

[3] Note that $\mu$ and $\sigma_{\rm T}$ in the likelihood (Eq. (4)) used in Eq. (A4) depends on $f$.

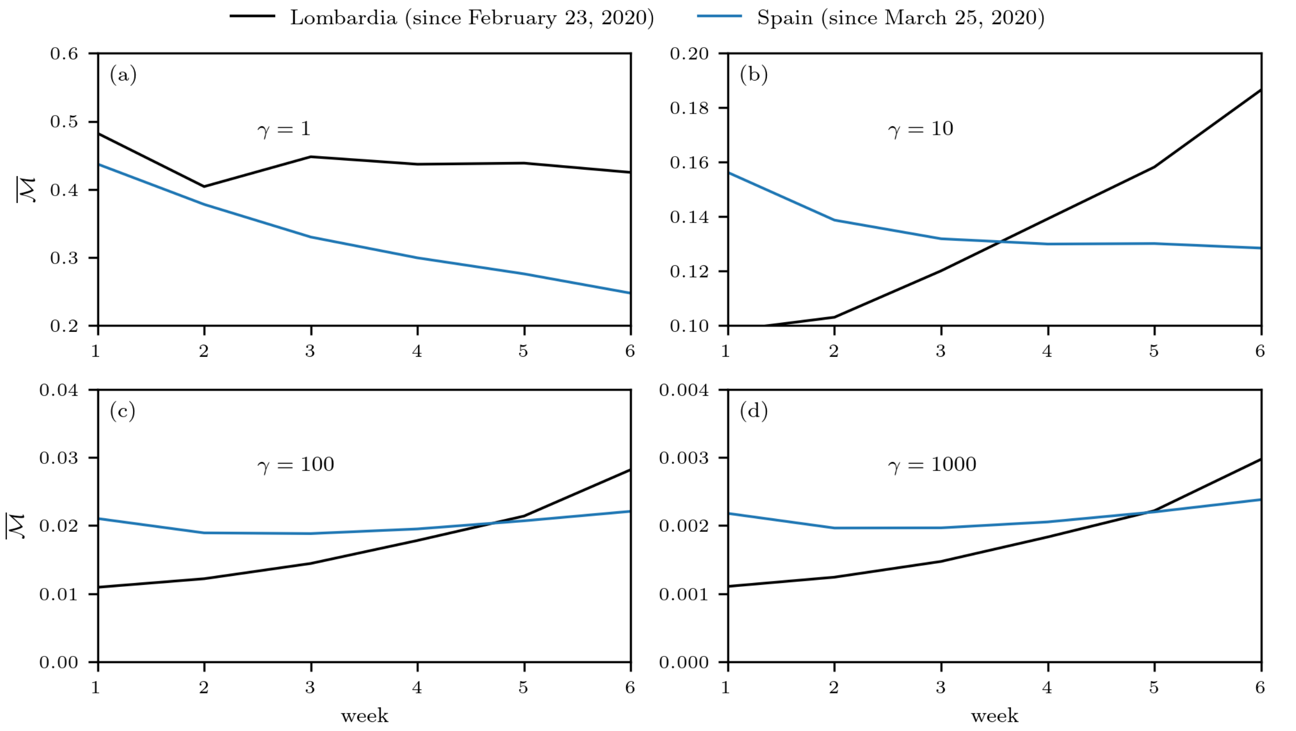


FIG. A3. **Evolution of resolved mortality.** We show the evolution of $\overline{\mathcal{M}}(t)$ for different values of effective recovery rates of unreported cases $\gamma$. The data are derived from Refs. [24, 27].

**Modeling of resolved mortality**

In Fig. A3, we show the evolution of $\overline{\mathcal{M}}$ for Spain and Lombardia, using different effective recovery rates of unreported cases $\gamma$. We compute $\overline{\mathcal{M}}$ according to Eq. (8) and use excess mortality data of Fig. 1 to determine $\bar{D}_{\mathrm{e}}$. The corresponding data for confirmed recovered and deceased individuals, $R_{\mathrm{c}}$ and $D_{\mathrm{c}}$, is taken from Ref. [28]. Current estimates of the IFR are $0.1 - 1.5\%$ [35–37]. To obtain a value of $\overline{\mathcal{M}}$ in a similar range, we vary $\gamma$ from $1 - 1000$ and find that $\overline{\mathcal{M}} \approx 0.1 - 1\%$ is consistent with $\gamma = 100 - 1000$.
